# Depletion of mRNA export regulator DBP5/DDX19, GLE1 or IPPK that is a key enzyme for the production of IP_6_, resulting in differentially altered cytoplasmic mRNA expression and specific cell defect

**DOI:** 10.1371/journal.pone.0197165

**Published:** 2018-05-10

**Authors:** Masumi Okamura, Yasutaka Yamanaka, Maki Shigemoto, Yuya Kitadani, Yuhko Kobayashi, Taiho Kambe, Masaya Nagao, Issei Kobayashi, Katsuzumi Okumura, Seiji Masuda

**Affiliations:** 1 Division of Integrated Life Sciences, Graduate School of Biostudies, Kyoto University, Kyoto, Kyoto, Japan; 2 Center for Molecular Biology and Genetics, Mie University, Tsu, Mie, Japan; 3 Department of Life Sciences, Graduate School of Bioresources, Mie University, Tsu, Mie, Japan; University of Toronto, CANADA

## Abstract

DBP5, also known as DDX19, GLE1 and inositol hexakisphosphate (IP_6_) function in messenger RNA (mRNA) export at the cytoplasmic surface of the nuclear pore complex in eukaryotic cells. DBP5 is a DEAD-box RNA helicase, and its activity is stimulated by interactions with GLE1 and IP_6_. In addition, these three factors also have unique role(s). To investigate how these factors influenced the cytoplasmic mRNA expression and cell phenotype change, we performed RNA microarray analysis to detect the effect and function of DBP5, GLE1 and IP_6_ on the cytoplasmic mRNA expression. The expression of some cytoplasmic mRNA subsets (e.g. cell cycle, DNA replication) was commonly suppressed by the knock-down of DBP5, GLE1 and IPPK (IP_6_ synthetic enzyme). The GLE1 knock-down selectively reduced the cytoplasmic mRNA expression required for mitotic progression, results in an abnormal spindle phenotype and caused the delay of mitotic process. Meanwhile, G1/S cell cycle arrest was observed in DBP5 and IPPK knock-down cells. Several factors that function in immune response were also down-regulated in DBP5 or IPPK knock-down cells. Thereby, *IFNβ-1* mRNA transcription evoked by poly(I:C) treatment was suppressed. These results imply that DBP5, GLE1 and IP_6_ have a conserved and individual function in the cytoplasmic mRNA expression. Variations in phenotype are due to the difference in each function of DBP5, GLE1 and IPPK in intracellular mRNA metabolism.

## Introduction

In eukaryotes, messenger RNA (mRNA) is transcribed in the nucleus by RNA polymerase II (RNAPII), and becomes messenger ribonucleoprotein (mRNP) by binding with a number of nuclear proteins for export to the cytoplasm [1–4]. mRNP undergoes the conformational change called “remodeling” when mRNP is exported to the cytoplasmic surface of the nuclear pore complex (NPC). The remodeling of mRNP at the cytoplasmic surface of NPC is required for the dissociation of mRNP from NPC into the cytoplasm.

The main factor in the remodeling is DBP5/DDX19, DEAD-box ATP-dependent RNA helicase [3]. DBP5 is localized on the cytoplasmic filament of NPC by interacting with the NPC component, Nup159 in *S*. *cerevisiae*, Nup214 in human [4,5]. The helicase activity is stimulated with the presence of GLE1 and inositol hexaphosphate (IP_6_) in *S*. *cerevisiae* [6–8]. Deletion of DBP5 in *S*. *cerevisiae* or knock-down of DBP5 in human cell line resulted in the accumulation of nuclear poly(A)^+^ RNA [9,10]. The binding of GLE1 and IP_6_ to DBP5 enhances its helicase activity. These findings implicate that the DBP5-GLE1-IP_6_ triplex also functions for the bulk poly(A)^+^ RNA export in human using helicase activity in DBP5. In addition to the role for mRNA export, DBP5 has a multiple roles including stabilization of ribosomal elongation and termination complexes, DNA damage response, and import of the SRF coactivator MKL1 [11–15].

A DBP5 regulator, GLE1, also has various functions in eukaryotic cells. There are two isoforms of GLE1: GLE1A and GLE1B [10]. GLE1A localizes in the cytoplasm and is used in the formation of stress granules [16]. In contrast, GLE1B localizes at the cytoplasmic surface of NPC and is used in mRNA export [10]. GLE1 is also used in the translation initiation and DBP5-GLE1-IP_6_ triplex plays a role in translation termination in *S*. *cerevisiae* [17]. Moreover, Gle1 regulates RNA binding of the DEAD-box helicase Ded1 in translation initiation[18,19]. Recently, it was also demonstrated that the localization of GLE1 in the centrosome plays a role in centrosome integrity [20].

IP_6_ is an inositol polyphosphate and highly conserved signaling molecule generated from IP_5_ by IPPK (also known as IPK1, IP5-2K) [21]. In addition to the function for mRNA export, IP_6_ has a role for translation [15]. IP_6_ bind to Ku subunits and specifically stimulates DNA-PK-dependent end-joining [22–24]. IP_6_ also bind to ADAR2 core and is required for RNA editing [25]. IPPK knock-down resulted in aberrant formation of left-right asymmetry because of the disruption of the Ca^2+^ signaling pattern in zebrafish [26].

Several studies show that the mutation of GLE1 is related to neurodegenerative diseases. The misspliced GLE1 caused by single nucleotide substitution leads to the genetic disease, lethal congenital contracture syndrome 1 (LCCS1) [27,28]. GLE1 mutation, named GLE1 Fin_Major_, decreased the efficiency of mRNA export and resulted in the disrupted development of schwann cell and neuron [29,30]. GLE1 deleterious mutation was also found in amyotrophic lateral sclerosis (ALS) patients [31]. This mutant GLE1 did not inhibit the mRNA export but has a tendency to form stress granules. It is known that protein aggregation and inefficient DNA repairing cause neurodegenerative diseases [32,33], therefore neurotoxicity should be taken into account when considering RNA metabolism and nucleocytoplasmic transport defects [34].

Although DBP5, GLE1 and IP_6_ function in mRNA export in an integrated manner, these three factors also reported to have multiple roles. We were interested in the finding that GLE1 showed a relation with neurodegenerative diseases but DBP5 and IP_6_ were not related to them. This prompted us to identify the exact effect on the cytoplasmic mRNA expression from each factor. In this study, we examined the cytoplasmic mRNA expression analysis using siRNA-mediated knock-down. We recovered the cytoplasmic RNA, and analyzed the array data and cell phenotypes to determine whether DBP5, GLE1 and IP_6_ have a general and unique role in the cytoplasmic mRNA expression. Results imply that DBP5, GLE1 and IP_6_ function as mRNA export regulators as well as exerting unique functions through regulating the unique target mRNA expression in the cytoplasm.

## Materials and methods

### Reagents and antibodies

BI2536, a Plk1 inhibitor, was purchased from Selleckchem (Houston, TX). Poly(I)-Poly(C) double-strand (poly(IC)), the TLR3 agonist, was obtained from GE Healthcare (Tokyo, Japan). Commercial antibodies used were as follows: mouse anti-FLAG M2 antibody (Sigma-Aldrich Japan, Tokyo, Japan), mouse anti-phospho histone H2A.X (γH2A.X) antibody (Merck Millipore, Darmstadt, Germany), mouse anti-IPPK polyclonal antibody (Sigma-Aldrich), mouse anti-β-Actin horseradish peroxidase (HRP)-conjugated antibody (Wako, Tokyo, Japan) and goat anti-lamin B antibody (Santa Cruz Biotechnology, Dallas, TX). DBP5 antiserum was obtained from Dr. Robin Reed. GLE1 antiserum was obtained from immunized rats. To perform an antiserum preparation, we carried out in strict accordance with the recommendations in the Guide for the Care and Use of Laboratory Animals of the Animal Committee in Kyoto University. This animal experiments were approved by the Committee on the Ethics of Animal Experiments of Kyoto University (Experiment permission number: Lif-K14004). All efforts were made to minimize suffering. Briefly, GST-GLE1(1–362 amino acids) fusion protein was produced in *E*. *coli* BL21 strain. The production of recombinant GST-GLE1N (1–362 amino acids) was induced with the addition of 0.5 mM IPTG for 4 h at 28 °C. Cells were pelleted with the centrifugation at 6000 × g for 10 min. The pelleted cells were resuspended with phosphate-buffered saline (PBS, pH, 7.2) containing 0.2 mM phenyl methyl sulfonyl fluoride (PMSF) and 1 mM dithiothreitol (DTT), and sonicated four times for 30 seconds on ice. The debris were pelleted by centrifugation at 8000 × g for 15 min. The clear lysate was transferred to a new tube and mixed with glutathione-fixed beads (GE Healthcare) overnight. The attached fusion protein GST-GLE1N was eluted with 30 mM glutathione in PBS containing 0.2 mM PMSF and 1 mM DTT. The eluate was dialyzed with PBS containing 0.2 mM PMSF and 1 mM DTT. The purity and concentration of the GST-GLE1N was determined with SDS-polyacrylamide gel electrophoresis (SDS-PAGE) using bovine serum albumin as a protein concentration standard. To obtain antiserum against GLE1, GST-GLE1N was immunized to wistar rats according to a previous report [35]. The titer of antiserum was confirmed by western blotting using HeLa nuclear extract and MBP-GLE1.

### Plasmid construction

3xFLAG-DBP5 expression vector was constructed by inserting full-length human DBP5 into AspI-XhoI sites of pcDNA5. GST-GLE1N was constructed by inserting the GLE1 N-terminal region into BamHI-XhoI sites of pGex6p2. H2B-EGFP expression plasmid was obtained from Dr. Matsumoto T., Kyoto University.

### Cell culture and stable cell lines

U2OS and HeLa cells were maintained in Dulbecco’s Modified Eagle’s Medium (DMEM) supplemented with 10% heat-inactivated fetal bovine serum (FBS) in a humidified atmosphere (5% CO_2_) at 37°C. HeLa cells stably expressing H2B-GFP was described previously [35].

U2OS cells stably expressing H2B-GFP were generated by H2B-EGFP expression plasmid transfection. Plasmid transfection was performed using Lipofectamine 2000 (Thermo Fischer Scientific, Yokohama, Japan). Briefly, U2OS cells were plated in 6-cm dishes in DMEM containing 10% FBS, a day before transfection, such that they were 60–70% confluent at the time of transfection. On the day of transfection, 5 μg of linearized H2B-GFP plasmid and 10 μL of Lipofectamine 2000 were incubated separately in 250 μL of Opti-MEM (Thermo Fischer Scientific). After 3 min of incubation at room temperature, the diluted plasmids and Lipofectamine 2000 were combined and incubated for an additional 20 min at room temperature. The DNA-Lipofectamine 2000 complexes were then added to each well. After overnight incubation, the medium was replaced with selective media (DMEM, 10% FBS, 100 μg/ml G418). The individual colony was checked by microscopy and the clones expressing EGFP in the nucleus were selected.

### siRNA and primers

The sequence of siRNAs used in this experiment is shown in S1 Table. Control #1, Control #2 and GLE1 #1 siRNA were purchased from Thermo Fischer Scientific. DBP5 #1, GLE1 #2, IPPK #1 and #2, PLK1 #1 and #2 and Kizuna (KIZ) #1 and #2 siRNAs were purchased from IDT Japan, Tokyo, Japan. PLK1 and Kizuna (KIZ) siRNAs were predesigned by IDT. The catalog number of PLK1 #1 is hs.Ri.PLK1.13.1, PLK1 #2 is hs.Ri.PLK1.13.2, KIZ #1 is hs.Ri.KIZ.13.1 and KIZ #2 is hs.Ri.KIZ.13.2.

### Immunofluorescence

U2OS cells (5–10% confluency) were inoculated on glass coverslips on a 12-well plate, and cultured for 24 h. The cells were then transfected with siRNA or plasmid as described below. Plasmid (0.4 μg) or siRNA (2.5 μl of 20 μM) was diluted with each 100 μl of OPTI-MEM medium. After mixing well, the diluted nucleic acid and 2 μl Lipofectamine 2000 were again mixed well and incubated for an additional 20 min at room temperature. The nucleic acid -Lipofectamine 2000 complex was then added to each well. After the transfection, cells were cultured for 48 h unless otherwise indicated, fixed in 4% formaldehyde in PBS and permeabilized with 0.1% Triton X-100 in PBS (PBS-T). After washing with PBS three times, the cells were blocked with 6% bovine serum albumin (BSA) in PBS for 1 h at room temperature, and the coverslips were incubated with primary antibody in PBS containing 2% BSA followed by secondary antibodies conjugated with Alexa-488 (Molecular Probes, Eugene, OR). Chromosomal DNA was stained with 4', 6-diamidino-2-phenylindole (DAPI).

### RNA fluorescence in situ hybridization

RNA fluorescence in situ hybridization was performed as described previously [35]. U2OS and/or HeLa cells (5–10% confluency) were inoculated on glass coverslips, and cultured for 24 h. The cells were then transfected with siRNA or plasmid as described in the section on immunofluorescence. After siRNA transfection, cells were cultured for 48 h, fixed in 10% formaldehyde in PBS for 20 min, and permeabilized in PBS-T for 10 min. The cells were then washed with PBS three times for 10 min and once with 2 × SSC for 5 min, prehybridized with ULTRAhyb-Oligo Hybridization Buffer (Ambion, Austin, TX) for 1 h at 42 °C in a humidified chamber, and incubated with 20 pmol Cy3-labeled oligo-dT_45_ probe diluted in hybridization buffer overnight. The cells were washed for 20 min at 42 °C with 2 × SSC, 0.5 × SSC, and then 0.1 × SSC, respectively. Quantification of the nuclear and the cytoplasmic poly(A)^+^ RNA signals was performed using ImageJ software (https://imagej.nih.gov/ij/) according to the instructions.

### Western blotting

U2OS cells were fractionated into cytoplasmic fraction, nuclear extraction and insoluble pellets in the following way. The cells were centrifuged, mixed with three times the pellet volume of solution A (10 mM Hepes-KOH (pH, 7.9), 1.5 mM MgCl_2_, 10 mM KCl, 0.2 mM PMSF and 0.5 mM DTT), and suspended very carefully. The cells were then incubated for 10 min on ice, vortexed for 5 sec, and centrifuged at 10000 × g for 10 sec. The supernatant was collected as the cytoplasmic fraction. The pellet was added to the same volume of solution C (20 mM Hepes-KOH (pH 7.9), 25% glycerol, 420 mM NaCl, 1.5 mM MgCl_2_, 0.2 mM EDTA, 0.2 mM PMSF and 0.5 mM DTT). The nuclear fraction was extracted for 20 min on ice, and obtained by centrifugation at 10000 × g for 10 min. The protein content in each fraction was determined by the Bradford assay (Nacalai tesque, Kyoto, Japan). The protein samples were mixed with 4 × SDS buffer (190 mM Tris-HCl (pH 6.8), glycerol 40%, SDS 0.8%, 0.2% Bromophenol blue, 40 mM DTT) and boiled for 2 min, separated by SDS-PAGE, and then electro-transferred to PVDF membrane using a Bio-Rad Trans-Blot cell. The blotted PVDF membrane was blocked with 5% skim milk/PBS containing 0.1% Tween 20 for 1 h at room temperature and reacted with the primary antibody, which was diluted with Can Get Signal Solution 1 (Toyobo, Kyoto, Japan) with contiguous rotating at 4 °C overnight. Blots were washed three times with PBS containing 0.1% Tween 20 for 10 min, respectively, and incubated with HRP-conjugated secondary antibody diluted with Can Get Signal Solution 2 (Toyobo) with contiguous rotating at room temperature for 2 h. The blotted membranes were washed with PBS containing 0.1% Tween 20 for 10 min three times, respectively, reacted with chemiluminescence reagent (Millipore Darmstadt, Germany) and detected with LAS 4000 mini (GE Healthcare).

### Cell proliferation assay

U2OS cells were inoculated at 2.5 × 10^4^ cells in a 6-well plate. After culturing for 24 h, the cells were transfected with siRNA using Lipofectamine 2000. On the day of transfection, 5 μL of 20 μM siRNA and 5 μL of Lipofectamine 2000 were incubated separately in 250 μL of Opti-MEM (Invitrogen), respectively. After 3 min of incubation at room temperature, the diluted siRNA and Lipofectamine 2000 were combined and incubated for an additional 20 min at room temperature. siRNA-Lipofectamine 2000 complex was then added to each well. The cells were recovered by trypsinization and the cell numbers were counted at the time indicated.

### RNA isolation, reverse transcription and real-time PCR

U2OS or HeLa cells (5–10% confluency) were transfected with DBP5, GLE1 or IPPK siRNA and cultured for 48 h. The cells were recovered by trypsinization and treated with lysis buffer (20 mM Tris-HCl pH, 8.0, 200 mM NaCl, 1 mM MgCl_2_, 1% NP40) on ice for 5 min. The cytoplasmic fraction was isolated by brief spin. RNA in the cytoplasmic fraction was isolated by Sepasol-RNA I super G (Nacalai tesque, Kyoto, Japan) according to the manufacturer’s instructions. Complementary DNA was synthesized from total or cytoplasmic RNA (4 μg) using 100 U ReverTraAce (Toyobo) and random 9 primer according to the manufacturer’s instructions. Real-time PCR was performed with Thunderbird SYBR qPCR Mix (Toyobo) and analyzed on a Thermal Cycler Dice real-time system II (Takara, Kyoto, Japan). Primer sets and real-time PCR conditions for this analysis are described in S2 Table.

### Microarray

Whole human genome DNA microarray 4x44K v2 (Agilent Technologies) was used for array analysis. Procedures for array analysis were performed according to the manufacturer’s instructions. The probe set signals were calculated using the microarray scanner model 2505 (Agilent, Santa Clara, CA). The raw data of gene expression were normalized by LOESS regression via R software (https://www.r-project.org). The rank-product-generated gene lists cut at 50% false discovery rate were uploaded into the ingenuity pathway analysis (IPA) (Agilent) server as input data. Canonical pathway analysis was conducted via IPA. The distribution of at least 1.5-fold downregulated genes in DBP5, GLE1 or IPPK knock-down cells was compared with that in the total probe set by Fischer’s exact test (as done automatically by the software), respectively. Three independent array experiments were conducted. The microarray data were submitted to Gene expression omnibus (GEO; accession number: GSE100424).

### Cell cycle synchronization and cell cycle analysis

To analyze the cell cycle arrest, the cells were synchronized by double thymidine block. U2OS cells were plated at 15–20% confluency in a culture dish with 2 mM thymidine. After 24 h incubation, thymidine was removed by washing with PBS, and fresh DMEM containing 10% FBS was added. Cells were further cultured for 24 h, and thymidine was again added to a final concentration of 2 mM. After 20 h incubation, thymidine was removed and cells were washed with PBS, and fresh DMEM containing 10% FBS was added. After releasing the cell cycle, thymidine was removed at the time indicated. The cells were fixed with 75% ethanol. The cells in ethanol were kept at 4 °C overnight. After fixation by ethanol, cells were treated with staining solution 1+2 (solution 1: 100 μg/ml propidium iodide, 0.1% Triton X100 and 0.1 mM EDTA in PBS, solution 2: 2 mg/ml RNase A in PBS) for 30 min. For staining chromosomal DNA, an equal volume of solutions 1 and 2 was mixed immediately before use. The cell cycle of control and siRNA-treated cells was confirmed by measuring the chromosomal DNA content in each cell using Accuri C6 Flow Cytometer (BD Biosciences, San Jose, CA).

When cell cycle synchronization was performed in siRNA transfection conditions, 5 × 10^4^ U2OS cells were inoculated in a 6-well plate with DMEM containing 10% FBS and 2 mM thymidine. Thymidine was washed out with PBS 24 h later, and siRNA was transfected using Lipofectamine 2000 according to the section on immunofluorescence. Thymidine was added again after 24 h. The transfected cells were incubated for 20 h. Thymidine was washed out from the cell culture, and 40 ng/ml nocodazole was added. The cells were fixed with 75% ethanol 0 to 12 h after nocodazole addition. The remainder of the steps were analyzed by the same procedure as described above.

### Live cell imaging

HeLa cells expressing H2B-GFP (5–10% confluency) grown on 35-mm glass-bottom dishes (Greiner Japan, Tokyo, Japan) were transfected with siRNA and Lipofectamine 2000 according to the section on immunofluorescence. After the transfection of siRNA, the cells were cultured for 40 h. The live images of the cells were taken every 6 min using BioStation IM-Q (Nikon, Tokyo, Japan) at 37 °C in 5% CO_2_ in a humidified chamber for 16 h.

### Statistics

Statistical analyses were conducted by a two-sided paired t-test or one-way ANOVA followed by Dunnett’s test using R software (https://www.r-project.org), as indicated in the figure legends. A *p*-value < 0.05 was considered significant.

### poly(I:C) treatment

poly(I:C) was diluted to 3 mg/ml with PBS. HeLa cells (3 × 10^5^ cells) were spread on a 10-cm dish. After 24 h incubation, siRNA was transfected with Lipofectamine 2000 according to the section on immunofluorescence. The cells were incubated for 45 h and were treated with 30 mg/ml poly(I:C) transfected with Lipofectamine 2000. Three hours later, total RNA was extracted as described above.

## Results

### Specific RNA subsets were suppressed by DBP5, GLE1 or IPPK knock-down

**Fig 1:**
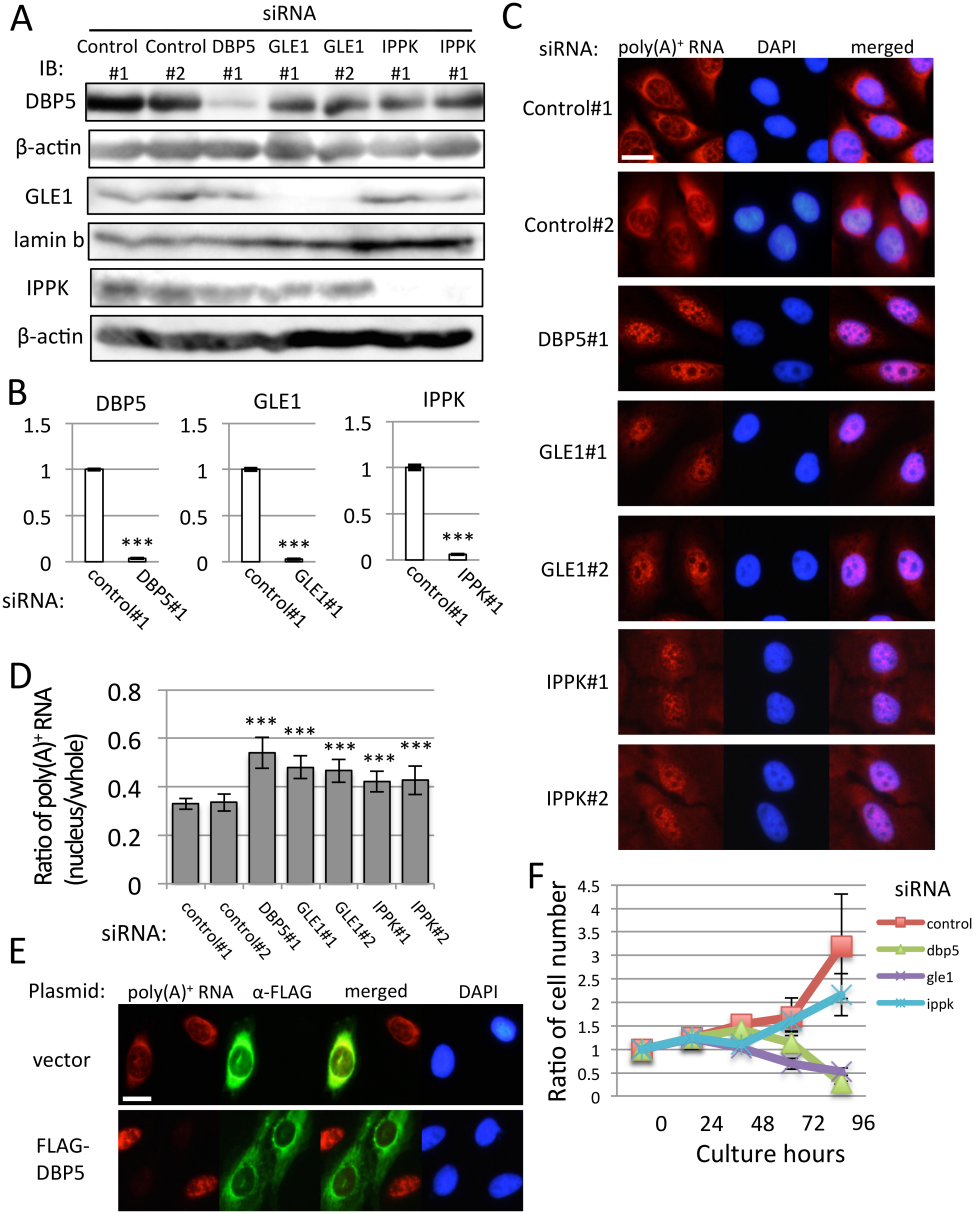
Nuclear accumulation of poly (A)^+^ RNA and deficiency of cell growth by knock-down of DBP5, GLE1 and IPPK. (A-D) U2OS cells were transfected by indicated siRNAs and cultured for 48 h. (A) Specific knock-down of DBP5, GLE1 and IPPK was confirmed by immunoblotting. We used the cytoplasmic fraction for the detection of DBP5, insoluble nuclear pellet for GLE1, and the nuclear fraction for IPPK. β-actin and lamin b were used as loading controls. IB: immunoblotting. (B) Real-time PCR analysis showed that each siRNA transfection significantly reduces cytoplasmic RNA of respective genes. Each value is the mean with standard deviation (SD) of three independent experiments. Error bars represent the SD. *p*-values were calculated by an unpaired student’s t-test by comparison with the control. (*** = *p*<0.001). (C) RNA-FISH reveals a nuclear accumulation of poly(A)^+^ RNA by DBP5, GLE1 and IPPK knock-down. Chromosome was counterstained with DAPI. Scale bar, 20 μm. (D) The ratio of the nucleus and whole-cell poly(A)^+^ RNA signals in C was quantified in each knocked-down cell. Each value is the mean with SD of three independent experiments. Error bars represent the SD. *p*-values were calculated using one-way ANOVA followed by Dunnett’s test by comparison with the control. (n = 20, *** = *p*<0.001, ** = *p*<0.01 and * = *p*<0.05). (E) U2OS cells transfected with FLAG-DBP5 expression plasmid or vector plasmid (pcDNA5) were cultured for 24 h, then transfected with DBP5 siRNA and cultured for 48 h. Immunofluorescence was performed using anti-FLAG M2 antibody. Scale bar, 20 μm. (F) The cell growth curve of U2OS cells transfected with indicated siRNAs. “0 h” represents the time when cells were spread. Cell numbers were counted every 24 h. siRNA were transfected 24 h after spreading. Each value is the mean with SD of three independent experiments. Error bars represent the SD.

To study the *in vivo* functions of DBP5, GLE1 and IPPK, siRNAs for these factors were designed. The siRNA for these factors were transfected using U2OS cells as described in the Materials and Methods. To confirm the efficiency of knock-down of each protein, we used specific antibodies against DBP5, GLE1 and IPPK. Using these antibodies, we checked whether each siRNA efficiently knocked down its corresponding protein by western blotting analysis (Fig 1A). We also examined the mRNA expression of each factor. As expected, each factor specifically inhibits its corresponding mRNA expression (Fig 1B). These three factors have a conserved function in the bulk mRNA export to the cytoplasm by forming a trimetric complex. Therefore, the inhibition of the nuclear export of bulk poly(A)^+^ RNA was examined in DBP5, GLE1 and IPPK knocked-down cells by RNA-FISH. The nuclear accumulation of poly(A)^+^ RNA was observed in all cases (Fig 1C). Measurement of mRNA in the nucleus was conducted by calculating the intensity of the nucleus and whole-cell poly(A)^+^ RNA signal using ImageJ. From the quantification of the poly(A)^+^ RNA signal of each knock-down sample, the knock-down of DBP5, the main machinery of the mRNA export, showed the most apparent poly(A)^+^ RNA accumulation in the nucleus (Fig 1D). The knock-down of GLE1 also accumulated poly(A)^+^ RNA in the nucleus. In contrast, the knock-down of IPPK showed moderate but clear accumulation of poly(A)^+^ RNA. To validate that the effect of DBP5 siRNA was specific to its corresponding mRNA, we performed rescue analysis by combining the knock-down of DBP5 and transfection of the rescue plasmid expressing siRNA-resistant DBP5 (Fig 1E) because we observed the phenotype from single siRNA against DBP5 (Fig 1A and 1C). DBP5, GLE1 and IPPK knock-down accumulated poly(A)^+^ RNA in the nucleus, suggesting that cell proliferation might be impaired. To examine this possibility, the cell number was counted every 24 h after siRNA transfection. As indicated in Fig 1F, the control siRNA treatment did not increase the cell number by 48 h because of the treatment of siRNA, and gradually increased the cell number by 96 h. The knock-down of DBP5 and GLE1 showed a similar growth phenotype by 48 h but failed to increase the cell number. In contrast, the knock-down of IPPK slowed down the growth rate compared with the control knock-down cells. The growth rate of knocked-down cells was well correlated with the accumulation of poly(A)^+^ RNA in the nucleus.

**Fig 2:**
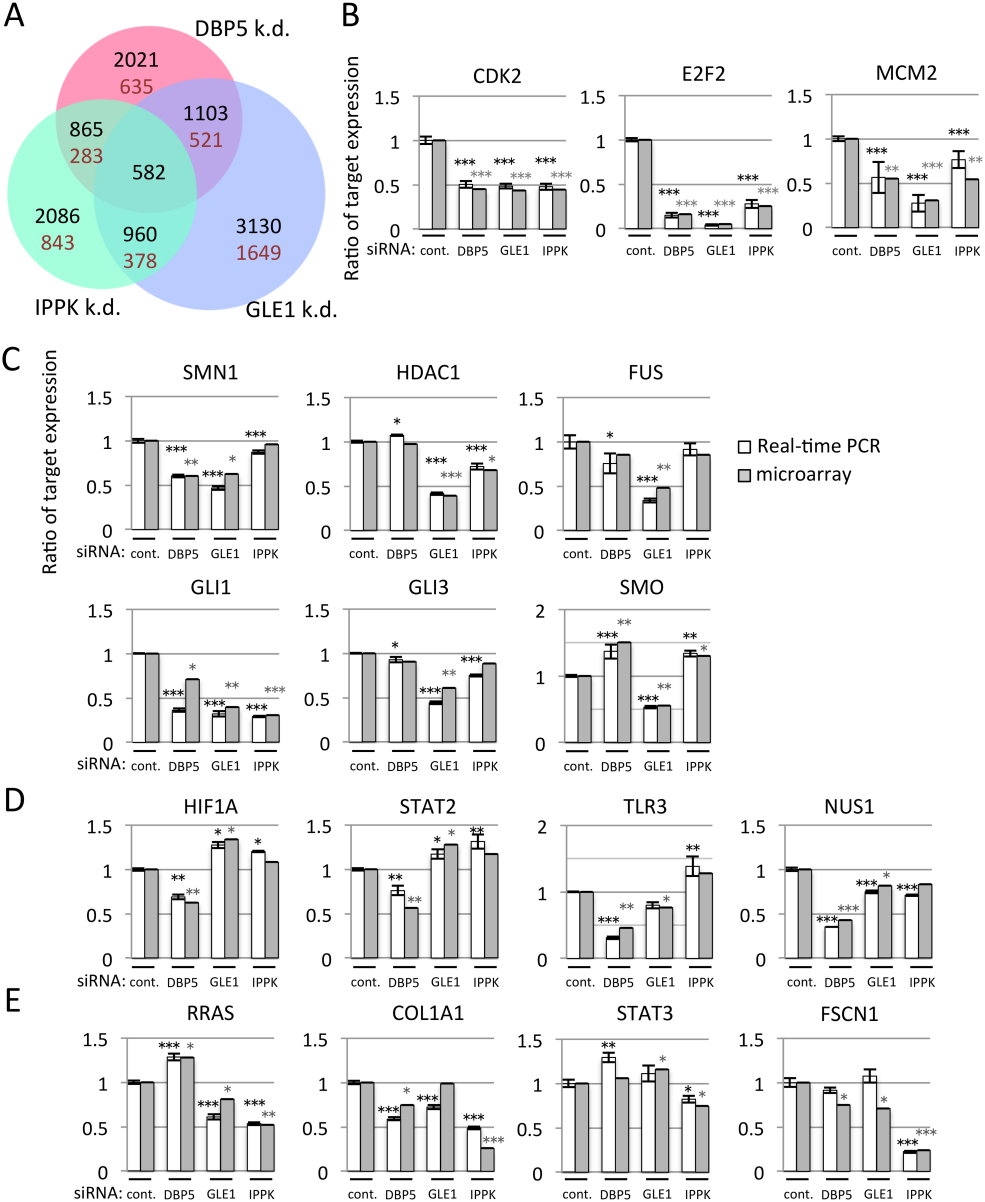
The knock-down of DBP5, GLE1 or IPPK affects the different subsets of cytoplasmic mRNA expressions. (A) The Venn diagram represents cytoplasmic transcripts reduced at least 1.5-fold in DBP5, GLE1 or IPPK knock-down cells. There were 30,412 probe sets on the array chip. The number in each circle indicates the number of genes detected. Black numbers: number of downregulated genes including overlapped part. Brown numbers: number of downregulated genes except overlapped part. (B-E) Validation of microarray data by real-time PCR. RNA was isolated from cytoplasm of U2OS cells transfected with indicated siRNA. White bar: the detected value of real-time PCR. Gray bar: the detected value of microarray. Each value is the mean with SD of three independent experiments. Error bars represent the SD. *p*-values were calculated by one-way ANOVA followed by Dunnett’s test by comparison with the control. (*** = *p*<0.001, ** = *p*<0.01 and * = *p*<0.05). (B) The cytoplasmic mRNAs of *CDK2*, *E2F2* and *MCM2* were decreased in common among DBP5, GLE1 and IPPK knocked-down cells. (C-E) GLE1, DBP5 and IPPK knock-down decreased the cytoplasmic mRNA. (C) GLE1, (D) DBP5 or (E) IPPK.

The depletion of each factor caused the accumulation of poly(A)^+^ RNA in the nucleus. However, the intensity of poly(A)^+^ RNA accumulation and the growth defect were different in each factor. To examine whether these factors have an identical role in overall cytoplasmic mRNA expression, we fractioned the cytoplasmic RNA (S1 Fig) and performed microarray analysis using whole human genome DNA microarray 4x44K v2. Each array data was validated by cluster analysis (S2 Fig). To compare the gene expression profiles and to focus on genes particularly susceptible to knock-down of DBP5, GLE1 or IPPK, the distribution of cytoplasmic levels for mRNAs was normalized. Efficient knock-down of individual mRNA (approximately 94% (DBP5), 72% (GLE1) and 73% (IPPK) reduction) was confirmed by microarray analyses. When a threshold was set at 1.5-fold reduction, microarray data indicated that the cytoplasmic levels of mRNAs from 2021, 3130 and 2086 genes were decreased in DBP5, GLE1 and IPPK knocked-down cells, respectively (Fig 2A). Among them, 582 genes decreased in common.

To validate the array analysis, we performed real-time PCR with selected genes and compared them with microarray data (Fig 2B–2E). We first analyzed the cytoplasmic mRNA expression of several cell cycle-related genes that are commonly downregulated by these factors (Fig 2B). They are clearly downregulated by knocking down each factor. We also validated the gene expressions that are especially affected by GLE1 (Fig 2C), DBP5 (Fig 2D) and IPPK (Fig 2E). From these data, we conclude that the microarray data were well correlated with real-time PCR results.

To observe the predisposition of gene number and characteristics affected by the knock-down of these factors, threshold set different stringent criteria at 1.5, 2 and 3-fold down-regulation was shown in S3–S5 Figs, respectively. In general, GLE1 knock-down most apparently affected the number of genes. The ratio of commonly down-regulated genes in total affected genes was decreased when a threshold was set more stringently, by contrast, that of uniquely down-regulated genes was increased in GLE1 and IPPK knock-down cells (S3 Table). DBP5 knock-down showed s similar direction. Considering that DBP5, GLE1 and IPPK have conserved roles in mRNA export and partly in translation, and multiple roles for mRNA metabolism, these results imply that the cytoplasmic mRNA expression by these three factors may be largely regulated by unique function of each factor. This possibility is further supported by the finding that the up-regulated genes by the knock-down of these factors were also observed (S6–S8 Figs). Up-regulation of the cytoplasmic mRNA expression was actually observed in mRNA expression in SMO by real-time PCR (Fig 2C). These results suggest that the functions of DBP5, GLE1 and IPPK overlap to some extent and these three factors also differentially regulate the cytoplasmic mRNA expression of particular species.

Even observing both effects for the cytoplasmic mRNA expression, the knock-down of these factors accumulated poly(A)^+^ RNA in the nucleus and reduced the cell proliferation, we focused on the genes down-regulated by these factors for further analysis. To validate the reduction of cytoplasmic mRNA expression was caused by the accumulation of the nuclear fraction by depleting DBP5, GLE1 or IPPK, the cytoplasmic and the nuclear mRNA of selected genes were analyzed by real-time PCR. The results indicated that some mRNA species were increased in the nucleus in one factor depleted condition, but other species were not (S9 Fig). We then examined the possibility that mRNA might be degraded in the nucleus when it is retained in the nucleus. To examine this possibility, we co-depleted RRP45, a component of exosome, with DBP, GLE1 or IPPK. Some mRNAs were affected by co-depletion with RRP45, implicating that the knock-down of DBP5, GLE1 or IPPK (especially in GLE1 depletion) seemed to activate the nuclear exosome activity to accelerate the specific degradation of mRNA like E2F2 and HDAC1 (S9 Fig). These results imply that the cytoplasmic mRNA expression in each gene is probably regulated by multiple mechanism including mRNA export and post-transcriptional degradation depending on each mRNA. We, therefore, performed the cell phenotype analysis rather than the further mechanistic analysis of the cytoplasmic and the nuclear mRNA expression.

To investigate whether downregulated genes in knock-down cells were functionally associated with particular cellular processes, we classified the downregulated genes by ingenuity pathway analysis (IPA) by calculating the *p*-value (S4 and S5 Tables). In IPA, we used canonical pathway analysis. This revealed that cell cycle-related genes were enriched in common by the knock-down. In contrast, genes regulated especially by GLE1 include damage response to the chromosome; those by DBP5 and IPPK include inflammatory responses (S4 Table).

### GLE1 knock-down leads to M-phase progression defect

**Fig 3:**
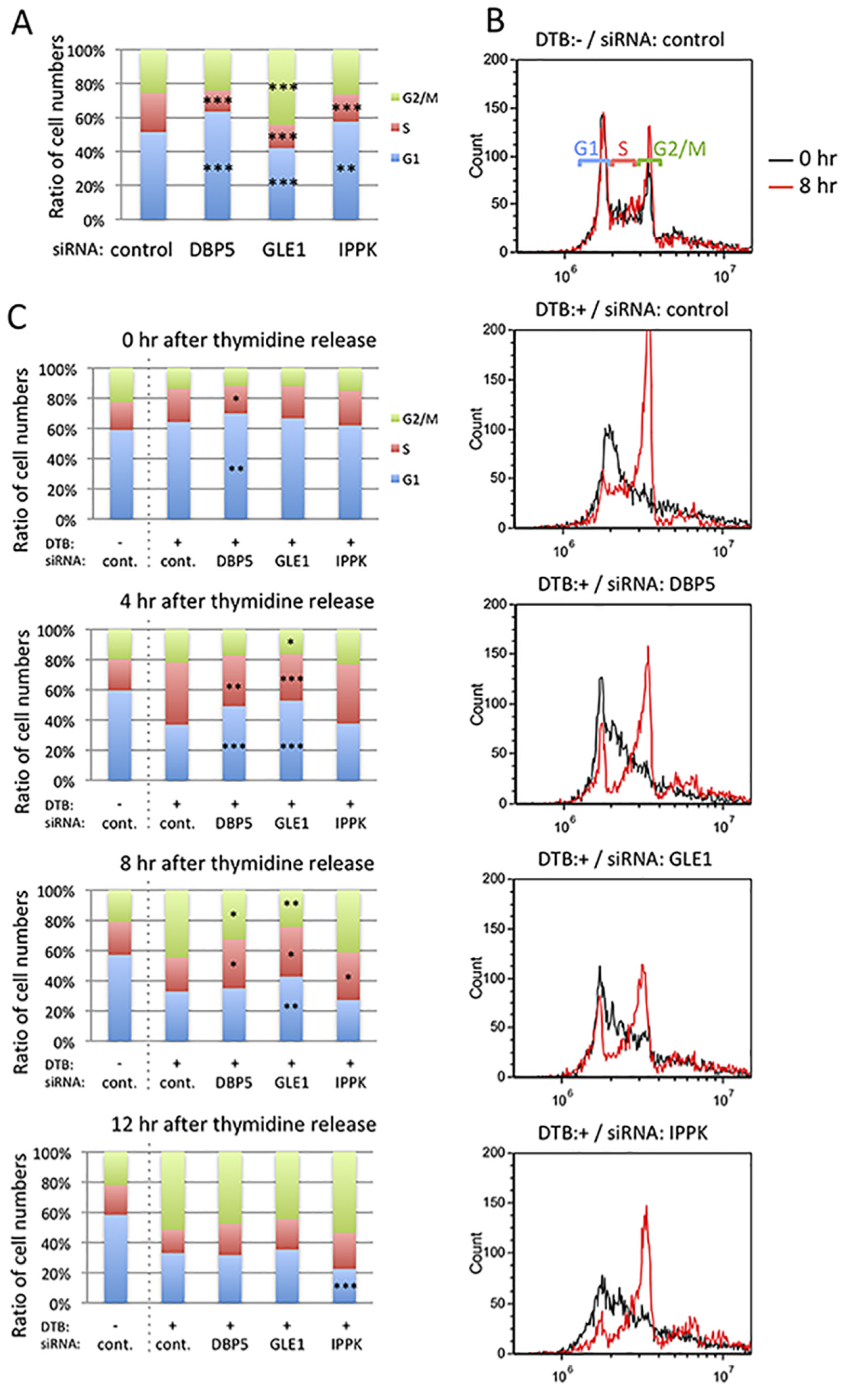
G1/S arrest increases by DBP5 and IPPK knock-down, and G2/M arrest increases by GLE1 knock-down. (A) The cell cycle phases of DBP5, GLE1 and IPPK knocked-down cells were investigated by flow cytometry. Each value is the mean with SD of three independent experiments. Light green color indicates G2/M phase. Red color indicates S phase. Blue color indicates G1 phase. *p*-values were calculated by one-way ANOVA followed by Dunnett’s test by comparison with the control. (*** = *p*<0.001 and ** = *p*<0.01). (B-C) Comparison among cells transfected with control siRNA without cell cycle synchronization, and cells transfected with control, DBP5, GLE1 or IPPK siRNA in cell cycle synchronization condition by the double thymidine block. DTB: double thymidine block. (B) Histograms show the cell number temporal change of each cell cycle phase. Black line: 0 h after thymidine release. Red line: 8 h after thymidine release. (C) Bar histograms represent the mean of three independent experiments. Light green color indicates G2/M phase. Red color indicates S phase. Blue color indicates G1 phase. *p*-values were calculated using an unpaired student’s t-test (*** = *p*<0.001, ** = *p*<0.01 and * = *p*<0.05).

As IPA indicated that the knock-down of these three factors might influence the cell cycle, we measured the cell cycle of cells treated with siRNAs against these factors. Phases of the cell cycle were indicated after 48 h of siRNA treatment (Fig 3A). The results clearly show that the S phase is decreased by each siRNA treatment. Conversely, the G1 phase is increased in DBP5 and IPPK knocked-down cells, and the G2/M phase is increased in GLE1 knocked-down cells.

We speculate that the decreased S-phase population in the knock-down of each factor resulted in the checkpoint block in the G1/S phase. To elucidate this possibility, we performed synchronization of the cell cycle by double thymidine block. The cell cycle was arrested at the end of the G1 phase by the excess amount of thymidine, thymidine was removed from the culture media to release the cell cycle, then nocodazole was added to keep the cell cycle at the M phase. The cells were fixed with 75% ethanol after releasing the cell cycle at 8 h, and the population of each cell phase was measured (Fig 3B). Treatment using double thymidine block resulted in most of the cell cycle being arrested in the G1 phase (black line). After releasing the cell cycle at 8 h by removing thymidine, cells in the M phase were markedly increased in control siRNA-treated cells. In contrast, the cells treated with DBP5 and IPPK knock-down showed a definite increase in the M phase, but the increase in the M-phase population was small compared with control siRNA-treated cells. In GLE1 knocked-down cells, the population of M-phase cells was increased but the M-phase rate was less than the others. This result indicates that the knock-down of DBP5, GLE1 and IPPK inhibits passing through the G1/S checkpoint. This is also supported by the result shown in Fig 3C of the cell cycle status time course after release from the G1/S checkpoint.

**Fig 4:**
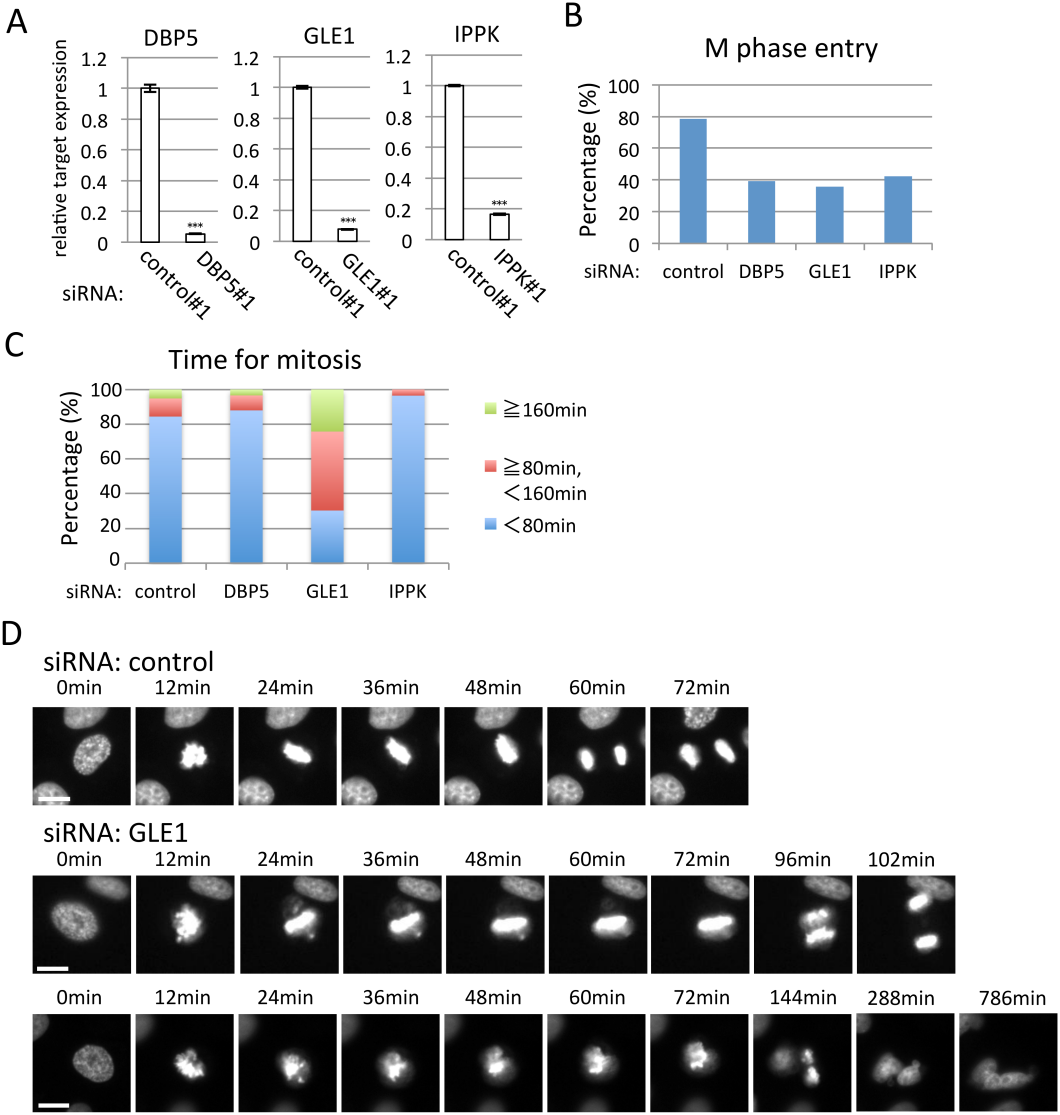
GLE1 knock-down results in mitotic progression defects. (A) Real-time PCR analysis using HeLa cells expressing H2B-GFP. Transfection of indicated siRNA significantly reduces cytoplasmic mRNA of respective gene. Each value is the mean with SD of three independent experiments. Error bars represent the SD. *p*-values were calculated an unpaired student’s t-test by comparison with the control. (*** = *p*<0.001). (B) The ratio of cells that could enter into the M phase during the time-lapse observation. The numbers of cells counted were 111, 66, 75 and 71 in control, DBP5, GLE1 and IPPK siRNA-treated cells, respectively. (C) The percentage of M-phase cells taking more or less than 80 min for mitosis. The numbers of cells counted were 96, 33, 58 and 28 in control, DBP5, GLE1 and IPPK siRNA-treated cells, respectively. (D) Representative successive live cell images for the indicated siRNA-transfected cells. Cells were observed 40–57 h after siRNA transfection, and the time was measured from M-phase progression by analyzing the recordings. Scale bar, 20 μm.

GLE1 knock-down increased the G2/M phase (Fig 3A) prompted us to observe M-phase progression using time-lapse microscopy. To perform time-lapse analysis, we used the HeLa cell line stably expressing H2B-GFP. Before starting the analysis, we confirmed that knock-down of each factor clearly depressed the cytoplasmic target mRNA expression in the HeLa cell line stably expressing H2B-GFP (Fig 4A). The population of cells entering into the M phase because of the knock-down of DBP5, GLE1 or IPPK was decreased compared with the control knocked-down cells (Fig 4B). The time to finish the M phase was mostly within 80 min in control cells (Fig 4C). The cells treated with DBP5 and IPPK siRNAs were also similar. In contrast, GLE1 knocked-down cells mostly took more than 80 min. This result implies that GLE1 knock-down especially downregulated the gene(s) required for M-phase progression. To see the detailed phenotype, we analyzed the imaging data and found that cells treated with GLE1 siRNA showed that the chromosome could not align but some chromosomes were scattered (Fig 4D and S1–S3 Movies), indicating that the lack of GLE1 influenced the spindle and/or centrosome formation. In U2OS cells stably expressing H2B-GFP, a delay in mitosis progression was also observed (S10 Fig and S4–S6 Movies), implying that the delay in M-phase progression was common in GLE1 knock-down cells.

**Fig 5:**
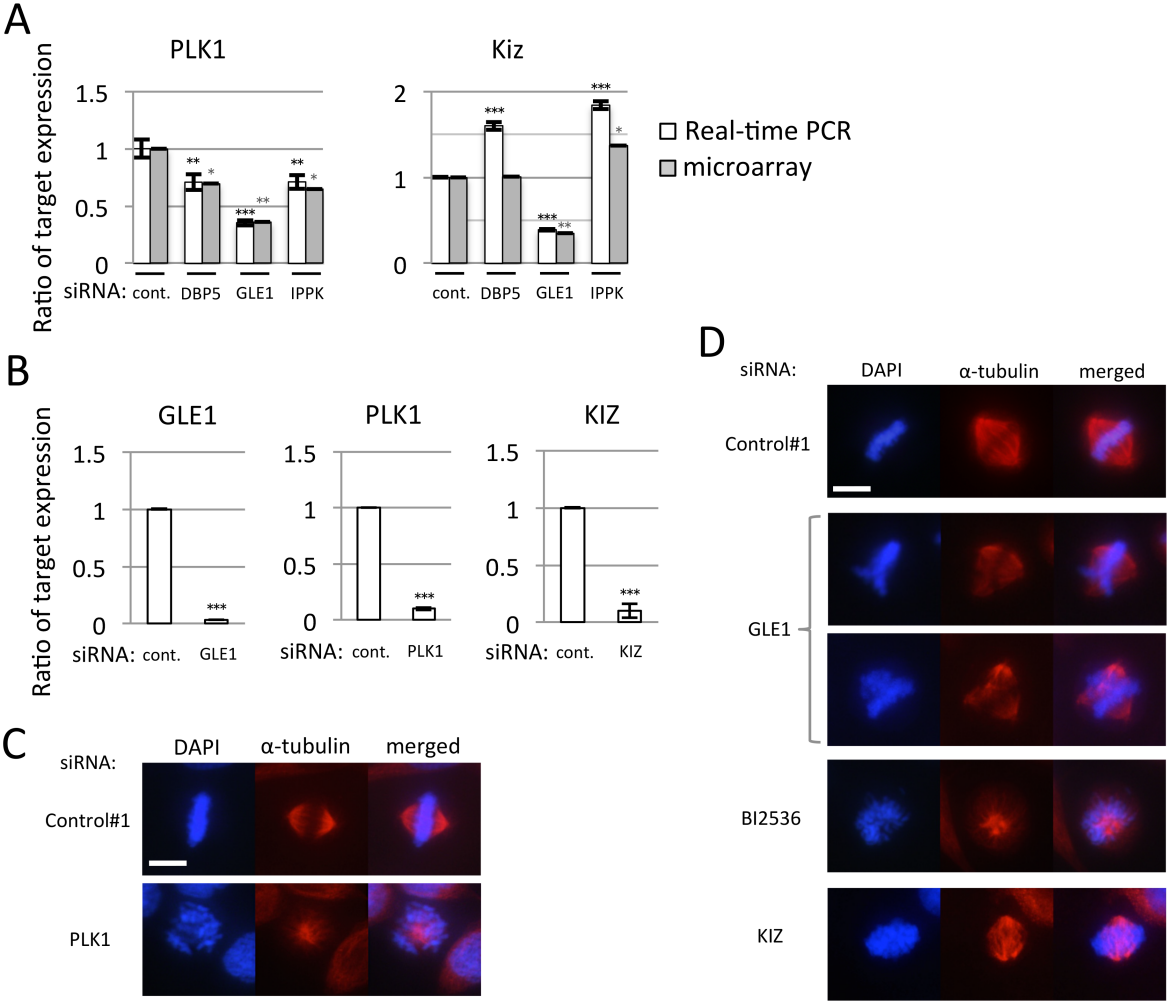
Treatment of siRNA against GLE1, PLK1 or KIZ caused abnormal spindles. Each value is the mean with SD of three independent experiments. Error bars represent the SD. *p*-values were calculated by one-way ANOVA followed by Dunnett’s test by comparison with the control. (*** = *p*<0.001, ** = *p*<0.01 and * = *p*<0.05). (A) Real-time PCR was performed using cDNA generated from cytoplasmic RNA of U2OS cells transfected with indicated siRNA. White bar: the detected value of real-time PCR. Gray bar: the detected value of microarray analysis. (B) Real-time PCR was performed to confirm the knock-down efficiency of GLE1, PLK1 and KIZ siRNAs. Cytoplasmic RNA of U2OS cells transfected with indicated siRNA was used to generate cDNA. Cells transfected with PLK1 siRNA were fixed after 24 h of culture, and GLE1 and KIZ were fixed after 48 h. (C-D) Staining of α-tubulin in cells treated with indicated siRNA. Cells were observed 24 h (C) or 48 h (D) after siRNA transfection. Chromosome was counterstained with DAPI. Scale bar, 20 μm.

The live cell imaging suggests that the knock-down of GLE1 resulted in the chromosome alignment defect and the M-phase progression delay. The microarray data followed by IPA analysis highlight the mitotic roles of polo-like kinases (S4 Table) as unique function of GLE1. We therefore picked two genes, PLK1 and Kizuna (KIZ) up as representative genes from the pathway of mitotic roles of polo-like kinases, because they are essential for the regulation of spindle bipolarity and the suppression of each factor caused the mitotic defect [36,37]. To validate the defect of M-phase progression caused by GLE1 knock-down, we observed the cytoplasmic expression of PLK1 and KIZ by real-time PCR. Real-time PCR as well as microarray results showed that the knock-down of DBP5 and IPPK reduced the expression of PLK1 but not KIZ (Fig 5A). GLE1 knock-down severely reduced the cytoplasmic expression of both mRNA, implying that the M-phase progression defect associated with GLE1 knock-down might be partly caused by the defect of cytoplasmic expression of PLK1 and KIZ. To examine this, we performed knock-down of PLK1 and KIZ and confirmed that these mRNAs were efficiently decreased by real-time PCR in HeLa cells (Fig 5B). The immunostaining of α-tubulin indicated that the lack of either PLK or KIZ resulted in the aberrant distribution of α-tubulin (Fig 5C and 5D). GLE1 depletion also caused a similar phenotype (Fig 5D). The treatment of PLK1 inhibitor also resulted in an aberrant distribution of α-tubulin. In addition, GLE1 knock-down affected the cytoplasmic mRNA expression of CDK1, CCNB1 and KIF11 affecting M phase progression. These results indicate that GLE1 deficiency specifically induces the aberrant centrosome and results in M-phase progression delay.

### GLE1 depletion attenuates DNA damage response most severely

**Fig 6:**
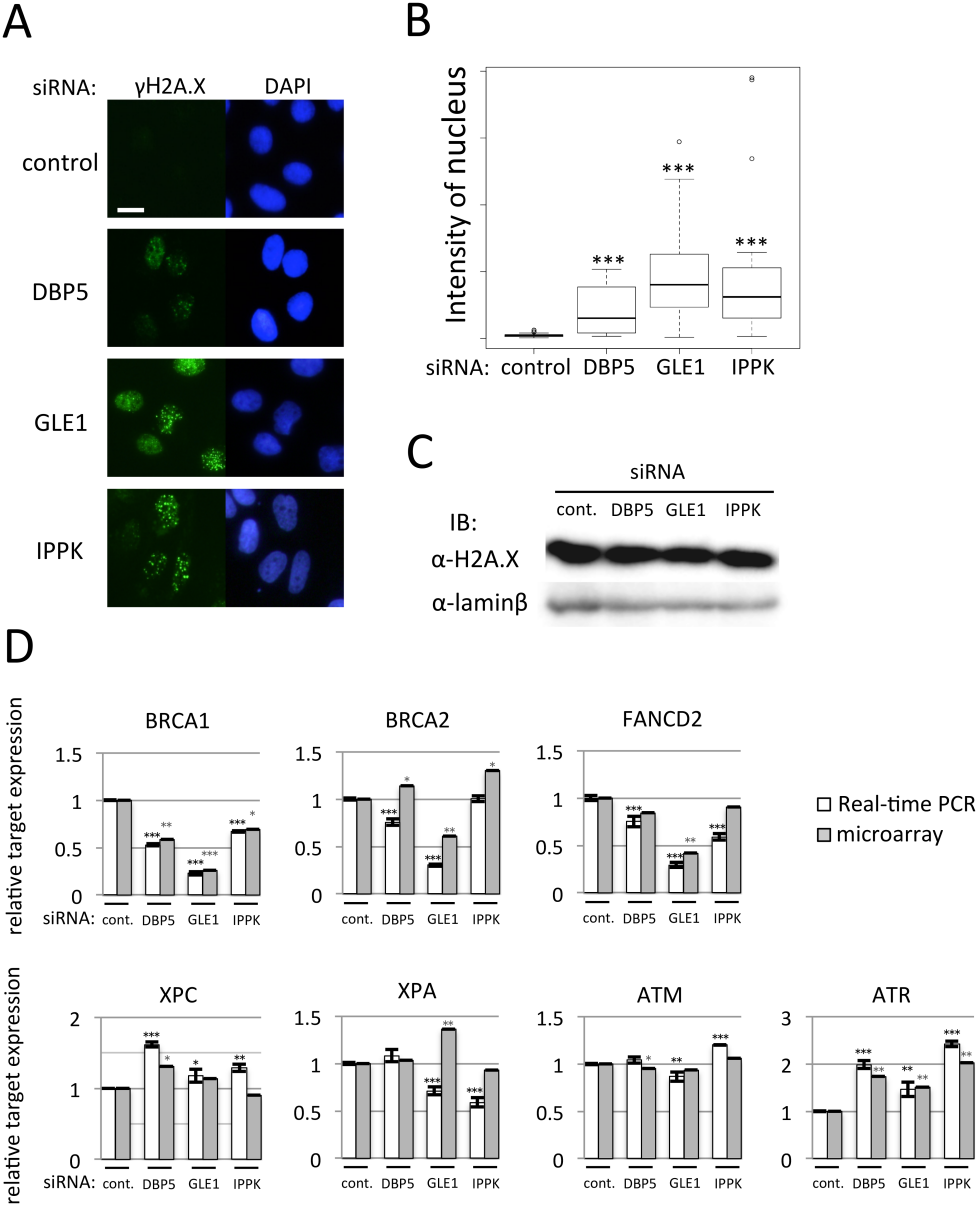
The knock-down of DBP5, GLE1 or IPPK alters DNA damage response. (A) U2OS cells transfected with siRNA of DBP5, GLE1 or IPPK were cultured for 48 h. After fixation, immunostaining was performed using anti-γH2A.X antibody. Chromosome was counterstained with DAPI. Scale bar, 20 μm. (B) The nuclear signal intensity of γH2A.X was quantified in each knock-down cell. Each value is the mean with SD of three independent experiments. Error bars represent the SD. *p*-values were calculated by one-way ANOVA followed by Dunnett’s test by comparison with the control. (n = 30, *** = *p*<0.001). (C) Immunoblotting against H2A.X was performed using the nuclear extract fraction of U2OS cells transfected with indicated siRNA. Lamin b was used as a loading control. (D) Real-time PCR was performed to detect cytoplasmic mRNA amounts of factors belonging to the DNA damage response category in IPA. Cytoplasmic total RNA was recovered from U2OS cells transfected with indicated siRNA. White bar: the detected value of real-time PCR. Gray bar: the detected value of microarray analysis. Each value is the mean with SD of three independent experiments. Error bars represent the SD. *p*-values were calculated by one-way ANOVA followed by Dunnett’s test by comparison with the control. (*** = *p*<0.001, ** = *p*<0.01 and * = *p*<0.05).

The microarray result implies that the knock-down of these factors also affects the DNA damage response (S4 Table). To examine whether the DNA damage was induced by the reduced expression of DBP5, GLE1 or IPPK, we stained phosphorylated H2A.X as a marker of double-strand DNA breaks using γH2A.X antibody. The depletion of these factors clearly induced γH2A.X positive foci in the nucleus (Fig 6A and 6B), indicating that the depletion of these factors induced the double-strand DNA breaks. GLE1 depletion showed the most prominent phenotype. The expression of total H2A.X was not changed with or without depletion of these factors (Fig 6C). To solve the cause of induction of γH2A.X positive foci, we measured the cytoplasmic mRNA expression in DNA damage sensor and DNA repair proteins by real-time PCR. As shown in Fig 6D, mRNA expressions of DNA damage protein affecting the single-strand DNA break, XPC, XPA and ATM, were not severely reduced. By contrast, the expressions of DNA repair factors BRCA1 and FANCD2, regulators for double-strand DNA breaks, were markedly reduced. The lack of GLE1 also greatly reduced the BRCA2 mRNA expression. The expression of ATR was instead increased by the knock-down of these factors. These results suggest that the depletion of DBP5, GLE1 or IPPK caused the reduction of DNA repair factor mRNAs in the cytoplasm and the DNA damage response delay.

### DBP5 or IPPK knock-down affects IFNB1 expression as an immune response

**Fig 7:**
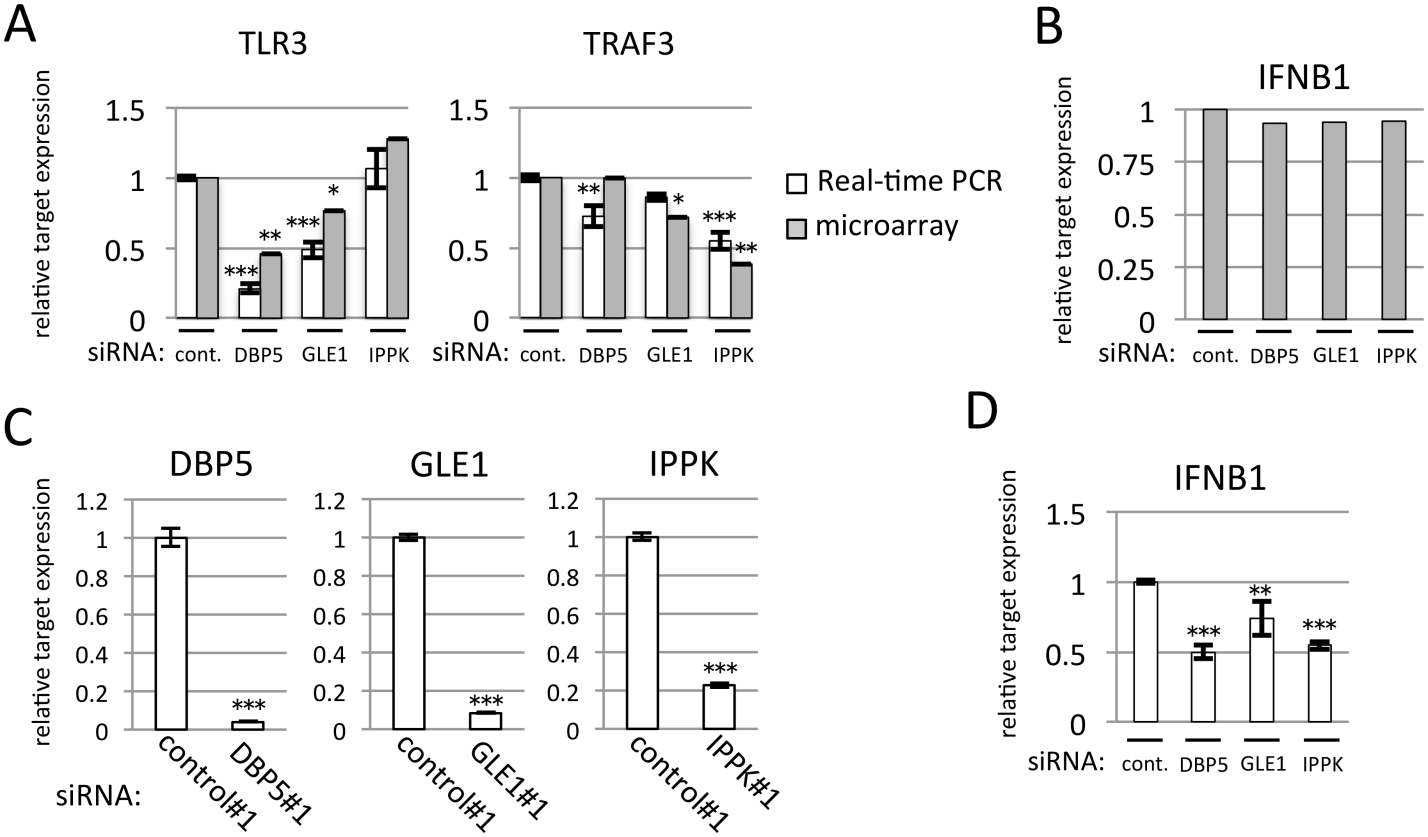
The knock-down of DBP5 or IPPK resulted in reduced induction of *IFNB1* caused by the poly(I:C) stimulation. Each value is the mean with SD of three independent experiments. Error bars represent the SD. *p*-values were calculated by one-way ANOVA followed by Dunnett’s test by comparison with the control. (*** = *p*<0.001, ** = *p*<0.01 and * = *p*<0.05). (A) Expression levels of *TLR3* and *TRAF3* mRNAs from microarray data were validated by real-time PCR. Cytoplasmic total RNA was recovered from HeLa cells transfected with indicated siRNA. White bar: the detected value of real-time PCR. Gray bar: the detected value of microarray analysis. (B) Microarray data of *IFNB1* mRNA level suggest that the knock-down of DBP5, GLE1 and IPPK does not impair the cytoplasmic mRNA expression of *IFNB1* at the basal expression level. (C) Real-time PCR was performed using cDNA generated from whole-cell RNA extracted by poly(I:C)-treated HeLa cells. Each siRNA transfection significantly reduces mRNA of an indicated gene. (D) *IFNB1* expression level detected by real-time PCR using HeLa cells. HeLa cells transfected by the indicated siRNA were cultured for 45 h, then treated with poly(I:C). Cells were fixed 3 h after poly(I:C) treatment, and RNA was isolated from whole-cell lysate.

We next examined the array results showing that DBP5 and IPPK depletion will affect inflammation and immune response genes (S4 Table). The knock-down of DBP5 reduced the cytoplasmic mRNA expression of TLR3, which is a member of the Toll-like receptor, and the knock-down of IPPK reduced the cytoplasmic mRNA expression of TRAF3, which associates with the TNF receptor and activates the immune response (Fig 7A). These results imply that the expression of IFNB1 is also reduced. However, the microarray data indicated that the cytoplasmic mRNA expression of *IFNB1* was not reduced (Fig 7B). This may reflect the fact that the basal expression level of *IFNB1* is quite low compared with the induced expression as a result of immune response. Therefore, we examined the induced *IFNB1* mRNA expression after the poly(I:C) transfection with or without the depletion of these factors. The method of *INFB1* mRNA induction by poly(I:C) has been described previously [38]. Cells were treated with DBP5, GLE1 or IPPK siRNA, and were then transfected with poly(I:C). Three hours later, total RNA was recovered and knock-down efficiency was confirmed to validate that transfection of poly(I:C) does not alter the reduction level of each factor (Fig 7C). We then detected the mRNA expression of *INFB1* by real-time PCR. As expected, the induction of *INFB1* mRNA was markedly reduced in DBP5 and IPPK knock-down cells. The knock-down of GLE1 also reduced the *INFB1* mRNA induction by poly(I:C) treatment but its reduction was weak compared with the reduction by DBP5 and IPPK knock-down.

## Discussion

DBP5, GLE1 and IPPK play a role in mRNA export, and are conserved from *S*. *cerevisiae* to human. In addition, these factors seem to have other functions besides mRNA export. However, the exact function of mRNA expression regulated by each factor in the cytoplasm has not been fully defined. In this study, we individually knocked down DBP5, GLE1 and IPPK, and analyzed the influence on mRNA expression in the cytoplasm. The depletion of each factor significantly increases the nuclear poly(A)^+^ RNA. We next examined the accumulated poly(A)^+^ RNA by the knock-down of each factor using microarray analysis. In the depletion condition, each factor was efficiently knocked down by real-time PCR and immunodetection of targets. The microarray results revealed that the total number of affected genes was roughly similar. However, part of genes were affected in common in all the knock-down samples, but others were not. In addition, the affected cytoplasmic mRNA expression seemed to be regulated by multiple mechanisms including mRNA export and post-transcriptional degradation. These results suggest that DBP5, GLE1 and IP_6_ exert common and unique roles on the cytoplasmic mRNA expression.

Pathway analysis using IPA reveals that affected pathway was largely different by the knock-down of these factors (S4 Table). Cell cycle related pathway was highlighted in commonly down-regulated genes. Signal related pathway was typical among uniquely down-regulated genes but signal pathway content was different in each knock-down. In addition, mitosis and DNA damage related pathway were observed in GLE1 knock-down cells. IPA analysis about 1.5 fold or more up-regulated genes highlighted to signal related pathway both in commonly and uniquely up-regulated genes but signal pathway content was again mostly different in each knock-down. From these analysis, DBP5, GLE1 and IP_6_ share common roles for some extent and have unique roles in the cytoplasmic mRNA expression. We note that IPPK catalyzes IP_6_ formation from IP_5_, and IP_6_ becomes the substrate to produce higher inositol polyphosphates like IP_7_ and IP_8_. We do not exclude the possibility that the unique mRNA expression in the cytoplasm by the depletion of IPPK might be partly derived from the lack of these higher polyphosphates. Even when the regulated genes were largely independent, inhibited cell growth was seen in all the knock-down samples. This would be partly because DBP5 and IPPK knock-down resulted in G1/S arrest and the accumulation of DNA damage, and the GLE1 knock-down-specific phenotype, the mitosis progression defect, resulted in the cell proliferation defect. The half-life of cell cycle related genes is short [39], suggesting that short-lived mRNAs were predominantly affected by the knock-down.

GLE1 is the responsible gene for LCCS1, a fetal neurodegenerative disease [27]. GLE1 mutation identified from LCCS1 patients deteriorates mRNA export efficiency [40]. This suggests that the pathogenesis of LCCS1 exists in the decreased mRNA expressions responsible for motoneurogenesis by GLE1 mutation. In this study, we confirmed that GLE1 knock-down reduces the cytoplasmic mRNA expression of some genes, like *FUS* and *HDAC1*(Fig 2C), which participate in neurodegenerative disease [41,42]. Furthermore, we detected the γH2A.X signal as an indicator of DNA damage. The GLE1 knock-down cells showed the highest-intensity γH2A.X signal. DBP5 and IPPK also reduced DNA repair factor mRNA expression more weakly than GLE1. The impaired DNA damage response led to neurodegeneration in the early developmental stage [43]. In addition to the abnormality of DNA damage response and mitosis, we detected the downregulation of *GLI3* and *SMO* in GLE1 knock-down cells. Both GLI3 and SMO have a pivotal role in Sonic hedgehog signaling. GLI3/Smo double mutants failed to stratify neural progeny in ventral spinal cord [44], and dysfunction of Sonic hedgehog signaling induces neuronal cell death [45]. The influence of miRNA is remarkable in motoneuronal disease as well as DNA damage response and mitosis [46]. A recent study showed the direct interaction of GLE1 with miR-376a-3p [47]. There is as yet no report of the contribution of DBP5, IPPK or IP_6_ to miRNA metabolism. Taking our findings together with previous findings, it might be possible to explain why GLE1 only has a relation with neurodegenerative diseases but DBP5 and IP_6_ are not related to them.

A recent study revealed that GLE1 in zebrafish localizes to the centrosome, and is essential for the integrity of microtubules [20]. In fact, the defect of mitosis progression was uniquely observed in GLE1-depleted cells. GLE1 depletion reduced the expression mitosis related genes like *PLK1*, *KIZ*, *CDK1*, *CCNB1* and *KIF11*. Rae1 (Gle2) plays a structural role in mitotic spindle assembly [48], suggesting that a similar non-nuclear export based role for GLE1 could explain its specific effects on mitosis. Knock-down of DBP5 or IPPK affected the TLR signaling pathway. The inhibition of the cytoplasmic *TLR3* mRNA expression, one of toll-like receptor family in human [49], was observed in DBP5 knock-down cells and *TRAF3* reduction in the cytoplasm, essential for type I INF including INFB, was instead observed by the knock-down of IPPK. The induction of *INFB1* mRNA stimulated by poly(I:C) treatment in the cytoplasm was probably suppressed by the reduction of a different gene expression between DBP5 and IPPK.

In summary, this study examined the function of DBP5, GLE1 and IPPK in the cytoplasmic mRNA expression and their impact on cell fate. As a result, we found both similarities and difference among these three factors. Estimating from the number of affected genes by the knock-down of DBP5, GLE1 or IPPK, GLE1 knock-down resulted in the widest alteration of mRNA expression in the cytoplasm. Some genes were affected in common but particular genes were uniquely affected by each protein knock-down, implicating that the function of DBP5, GLE1 and IPPK has specific role(s) in the cytoplasmic mRNA expression and the determination of cytoplasmic mRNA expression seemed to be more dependent on their unique roles than previously expected.

## Supporting information

S1 Fig Fractionation of the cytoplasmic and the nuclear total RNA. The fractionation of cytoplasmic and nuclear RNA was carried out as follows. The cells were recovered by trypsinization and treated with lysis buffer (20 mM Tris-HCl pH, 8.0, 200 mM NaCl, 1 mM MgCl_2_, 1% NP40) on ice for 5 min. The cytoplasmic RNA fraction was isolated by brief spin. RNA in the cytoplasmic fraction was isolated by Sepasol-RNA I super G (Nacalai tesque, Kyoto, Japan) according to the manufacturer’s instructions. The pellet was washed once with lysis buffer. The nuclear RNA was isolated using Sepasol-RNA I super G. U6 snRNA was used for the nucleus fraction specific RNA. tRNA was used for the cytoplasmic selective RNA. E2F8 mRNA was used to confirm that the fractionation was successfully performed.
(PNG)

S2 Fig Cluster analysis of microarray data. The data from RNA microarray experiments were grouped together and are connected by a series of branches. RNA samples transfected by siRNA formed the same group together. Red numbers: Approximately unbiased *p*-value. Green numbers: Bootstrap probability value.
(PNG)

S3 Fig The Venn diagram represents cytoplasmic transcripts reduced at least 1.5-fold in DBP5, GLE1 or IPPK knock-down cells. There were 30,412 probe sets on the array chip. Left panel: The total number in each circle indicates the number of genes detected. Right panel: The number in each part indicates the number of genes detected except for overlapped part.
(PNG)

S4 Fig The Venn diagram represents cytoplasmic transcripts reduced at least 2-fold in DBP5, GLE1 or IPPK knock-down cells. There were 30,412 probe sets on the array chip. Left panel: The total number in each circle indicates the number of genes detected. Right panel: The number in each part indicates the number of genes detected except for overlapped part.
(PNG)

S5 Fig The Venn diagram represents cytoplasmic transcripts reduced at least 3-fold in DBP5, GLE1 or IPPK knock-down cells. There were 30,412 probe sets on the array chip. Left panel: The total number in each circle indicates the number of genes detected. Right panel: The number in each part indicates the number of genes detected except for overlapped part.
(PNG)

S6 Fig The Venn diagram represents cytoplasmic transcripts increased at least 1.5-fold in DBP5, GLE1 or IPPK knock-down cells. There were 30,412 probe sets on the array chip. Left panel: The total number in each circle indicates the number of genes detected. Right panel: The number in each part indicates the number of genes detected except for overlapped part.
(PNG)

S7 Fig The Venn diagram represents cytoplasmic transcripts increased at least 2-fold in DBP5, GLE1 or IPPK knock-down cells. There were 30,412 probe sets on the array chip. Left panel: The total number in each circle indicates the number of genes detected. Right panel: The number in each part indicates the number of genes detected except for overlapped part.
(PNG)

S8 Fig The Venn diagram represents cytoplasmic transcripts increased at least 3-fold in DBP5, GLE1 or IPPK knock-down cells. There were 30,412 probe sets on the array chip. Left panel: The total number in each circle indicates the number of genes detected. Right panel: The number in each part indicates the number of genes detected except for overlapped part.
(PNG)

S9 Fig Comparison of the mRNA expression in the cytoplasm and the nucleus. A-D, The cytoplasmic (red color) and the nuclear (blue color) mRNA expression level were measured and normalized with PGK1 by real-time PCR. A, CDK2, B, E2F2, C, HDAC1, D, HIF1A, The cytoplasmic mRNA expression level in each condition was set as 1. Each value is the mean with standard deviation of three independent experiments. Error bars represent standard deviations. E-H, The level of mRNA in DBP5, GLE1 or IPPK depleted condition in the nucleus (blue color) was compared with those of the condition co-depleted with RRP45 (red color). E, CDK2, F, E2F2, G, HDAC1, H, HIF1A, The mRNA expression level in the nucleus in each factor depleted condition was set as 1. Each value is the mean with standard deviation of three independent experiments. Error bars represent standard deviations.
(PNG)

S10 Fig GLE1 knock-down resulted in mitotic progression delay in U2OS cells. (A) The ratio of cells that could enter into the M phase. (B) The percentage of M-phase cells taking more or less than 80 min for mitosis. The numbers of cells counted were 126, 20, 10 and 33 in control, DBP5, GLE1 and IPPK siRNA-treated cells, respectively. (C) Representative successive live cell images for the indicated siRNA-transfected cells. Cells were observed 40–57 h after siRNA transfection, and the time was measured from M-phase progression by analyzing the recordings. Scale bar, 20 μm.
(PNG)

S1 Movie Mitotic progression in HeLa cell. Movie of H2B-GFP HeLa cell transfected with control siRNA. One second corresponds to 60 min.
(AVI)

S2 Movie Mitotic progression in GLE1-depleted HeLa cell (A). Movie of H2B-GFP HeLa cell transfected with GLE1 siRNA (A) is shown. One second corresponds to 60 min.
(AVI)

S3 Movie Mitotic progression in GLE1-depleted HeLa cell (B). Movie of H2B-GFP HeLa cell transfected with GLE1 siRNA (B) is shown. One second corresponds to 60 min.
(AVI)

S4 Movie Mitotic progression in U2OS cell. Movies of H2B-GFP U2OS cell transfected with control siRNA is shown. One second corresponds to 120 min.
(AVI)

S5 Movie Mitotic progression in GLE1 depleted U2OS cell (C). Movies of H2B-GFP U2OS cell transfected with GLE1 siRNA (C) is shown. One second corresponds to 120 min.
(AVI)

S6 Movie Mitotic progression in GLE1 depleted U2OS cell (D). Movies of H2B-GFP U2OS cell transfected with GLE1 siRNA (D) is shown. One second corresponds to 120 min.
(AVI)

S1 Table The sequences of siRNAs in this study. (XLSX)

S2 Table Primer sets and annealing temperature in this study. (XLSX)

S3 Table Gene numbers and ratio affected by DBP5, GLE1 or IPPK knock-down. (XLSX)

S4 Table Gene categories which are affected mRNA expression in DBP5, GLE1 or IPPK knock-down cells. Genes down-regulated and up-regulated at least 1.5-fold in DBP5, GLE1 or IPPK knock-down cells were categorized by canonical pathway analysis in ingenuity pathway analysis. Ten categories are listed in order of their *p*-values.
(XLSX)

S5 Table Gene categories which are 1.5- and 2-fold affected mRNA expression including overlapping parts in DBP5, GLE1 or IPPK knock-down cells. Genes down-regulated and up-regulated at least 1.5- and 2-fold in DBP5, GLE1 or IPPK knock-down cells were categorized by canonical pathway analysis in ingenuity pathway analysis. Ten categories are listed in order of their *p*-values.
(XLSX)
