# Correction: Depletion of mRNA export regulator DBP5/DDX19, GLE1 or IPPK that is a key enzyme for the production of IP_6_, resulting in differentially altered cytoplasmic mRNA expression and specific cell defect

**DOI:** 10.1371/journal.pone.0220511

**Published:** 2019-07-25

**Authors:** Masumi Okamura, Yasutaka Yamanaka, Maki Shigemoto, Yuya Kitadani, Yuhko Kobayashi, Taiho Kambe, Masaya Nagao, Issei Kobayashi, Katsuzumi Okumura, Seiji Masuda

There are errors in Fig 1, “Nuclear accumulation of poly (A)+ RNA and deficiency of cell growth by knock-down of DBP5, GLE1 and IPPK,” panel E and caption. Please see the correct Fig 1 and correct caption here.

**Fig 1:**
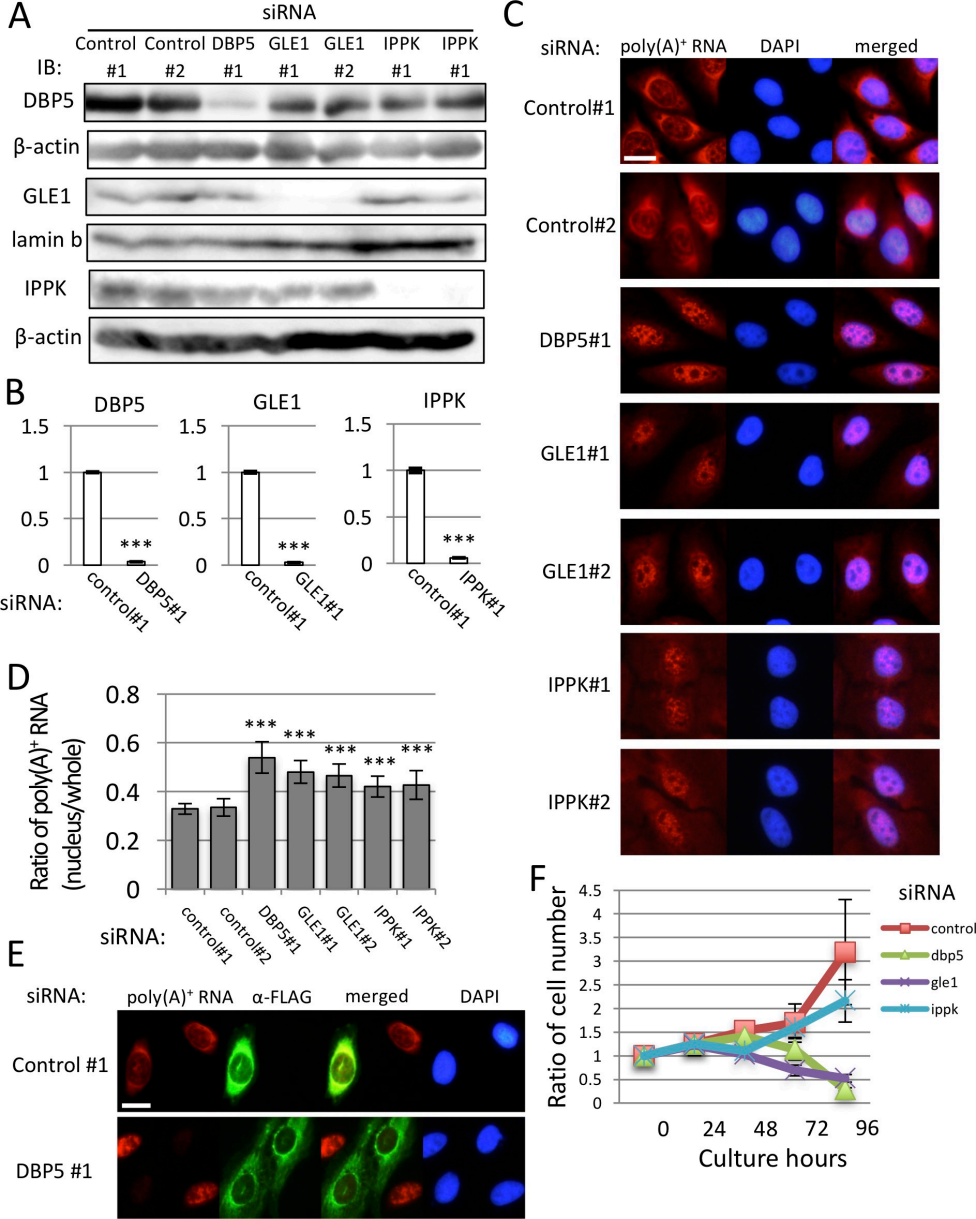
Nuclear accumulation of poly (A)^+^ RNA and deficiency of cell growth by knock-down of DBP5, GLE1 and IPPK. (A-D) U2OS cells were transfected by indicated siRNAs and cultured for 48 h. (A) Specific knock-down of DBP5, GLE1 and IPPK was confirmed by immunoblotting. We used the cytoplasmic fraction for the detection of DBP5, insoluble nuclear pellet for GLE1, and the nuclear fraction for IPPK. β-actin and lamin b were used as loading controls. IB: immunoblotting. (B) Real-time PCR analysis showed that each siRNA transfection significantly reduces cytoplasmic RNA of respective genes. Each value is the mean with standard deviation (SD) of three independent experiments. Error bars represent the SD. *p*-values were calculated by an unpaired student’s t-test by comparison with the control. (*** = *p*<0.001). (C) RNA-FISH reveals a nuclear accumulation of poly(A)^+^ RNA by DBP5, GLE1 and IPPK knock-down. Chromosome was counterstained with DAPI. Scale bar, 20 μm. (D) The ratio of the nucleus and whole-cell poly(A)^+^ RNA signals in C was quantified in each knocked-down cell. Each value is the mean with SD of three independent experiments. Error bars represent the SD. *p*-values were calculated using one-way ANOVA followed by Dunnett’s test by comparison with the control. (n = 20, *** = *p*<0.001, ** = *p*<0.01 and * = *p*<0.05). (E) U2OS cells transfected with FLAG-DBP5 expression plasmid were cultured for 24 h, then transfected with control or DBP5 siRNA and cultured for 48 h. Immunofluorescence was performed using anti-FLAG M2 antibody. Scale bar, 20 μm. (F) The cell growth curve of U2OS cells transfected with indicated siRNAs. “0 h” represents the time when cells were spread. Cell numbers were counted every 24 h. siRNA were transfected 24 h after spreading. Each value is the mean with SD of three independent experiments. Error bars represent the SD.
