# Supplementary figures and images for: Depletion of mRNA export regulator DBP5/DDX19, GLE1 or IPPK that is a key enzyme for the production of IP6, resulting in differentially altered cytoplasmic mRNA expression and specific cell defect
Source: PLoS One. 2018 May 10;13(5):e0197165. doi: 10.1371/journal.pone.0197165 (PMC5945018; doi:10.1371/journal.pone.0197165)

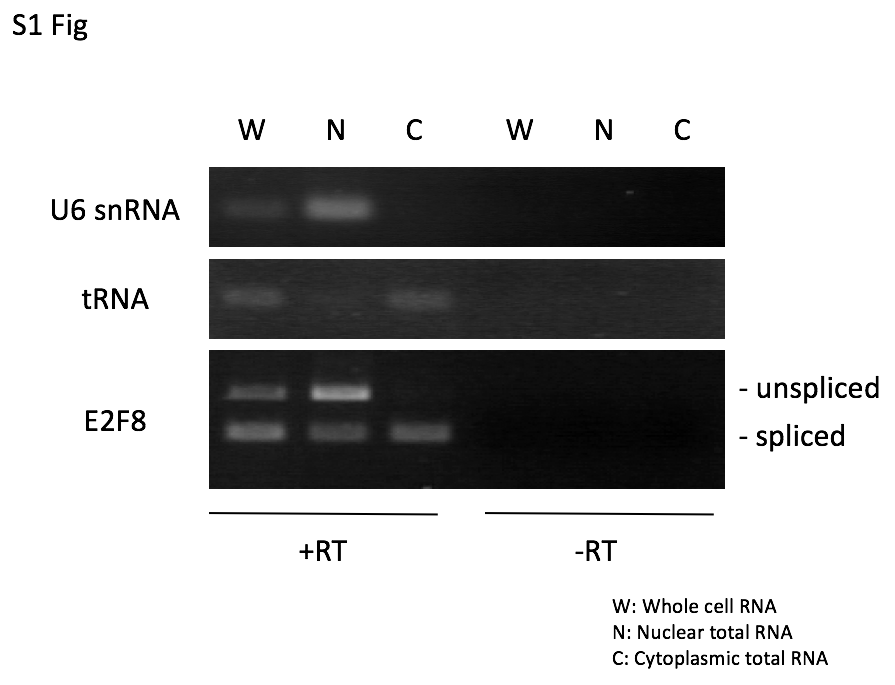

Supplement: S1 Fig — The fractionation of cytoplasmic and nuclear RNA was carried out as follows. The cells were recovered by trypsinization and treated with lysis buffer (20 mM Tris-HCl pH, 8.0, 200 mM NaCl, 1 mM MgCl2, 1% NP40) on ice for 5 min. The cytoplasmic RNA fraction was isolated by brief spin. RNA in the cytoplasmic fraction was isolated by Sepasol-RNA I super G (Nacalai tesque, Kyoto, Japan) according to the manufacturer’s instructions. The pellet was washed once with lysis buffer. The nuclear RNA was isolated using Sepasol-RNA I super G. U6 snRNA was used for the nucleus fraction specific RNA. tRNA was used for the cytoplasmic selective RNA. E2F8 mRNA was used to confirm that the fractionation was successfully performed. (PNG) [file pone.0197165.s001.png]

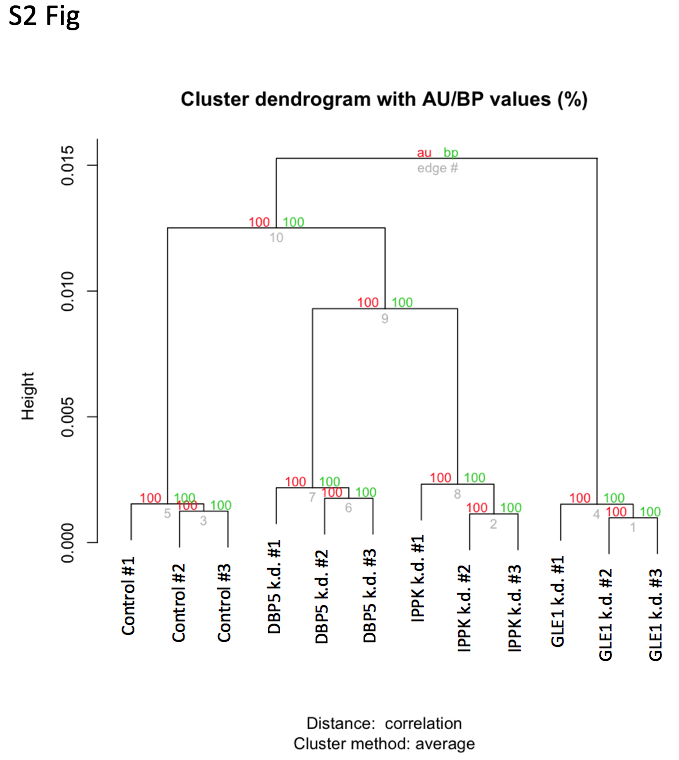

Supplement: S2 Fig — The data from RNA microarray experiments were grouped together and are connected by a series of branches. RNA samples transfected by siRNA formed the same group together. Red numbers: Approximately unbiased p-value. Green numbers: Bootstrap probability value. (PNG) [file pone.0197165.s002.png]

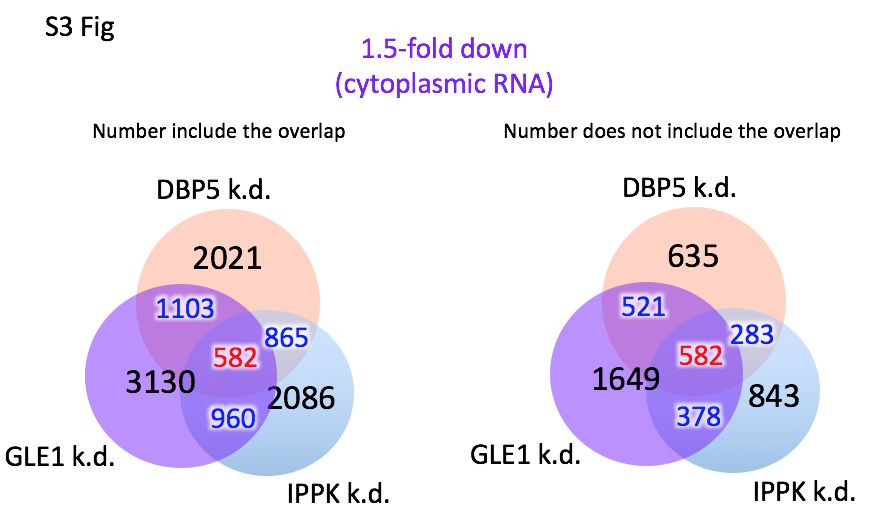

Supplement: S3 Fig — There were 30,412 probe sets on the array chip. Left panel: The total number in each circle indicates the number of genes detected. Right panel: The number in each part indicates the number of genes detected except for overlapped part. (PNG) [file pone.0197165.s003.png]

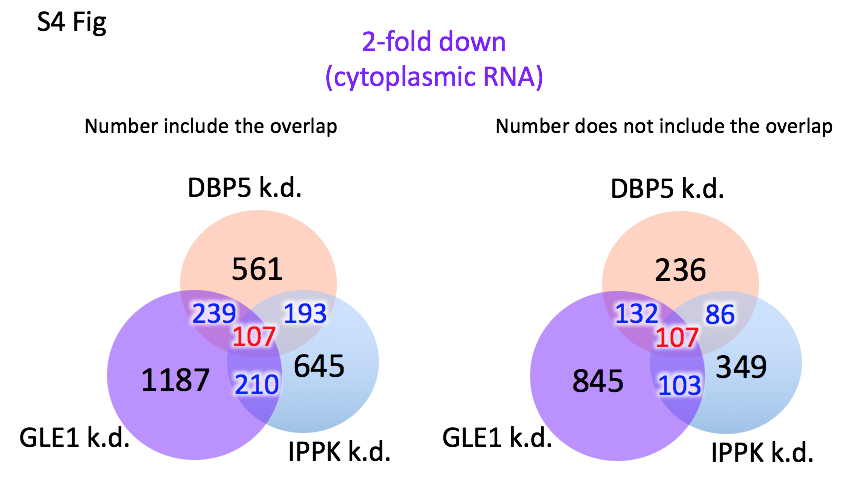

Supplement: S4 Fig — There were 30,412 probe sets on the array chip. Left panel: The total number in each circle indicates the number of genes detected. Right panel: The number in each part indicates the number of genes detected except for overlapped part. (PNG) [file pone.0197165.s004.png]

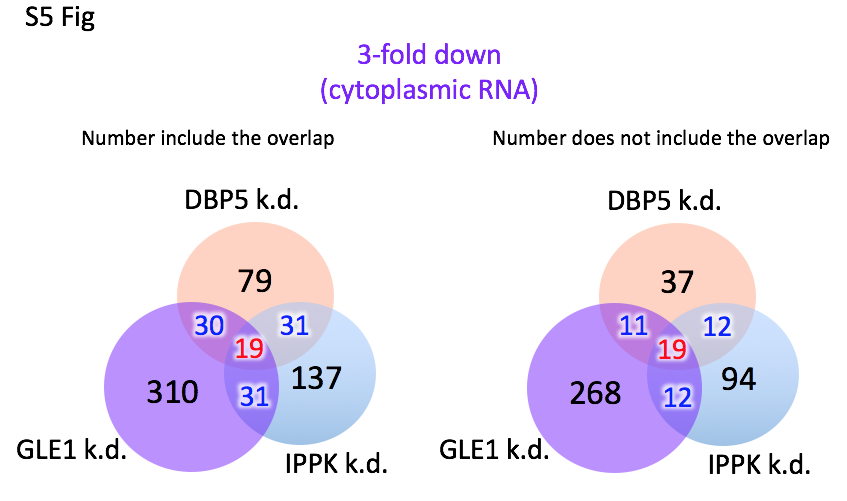

Supplement: S5 Fig — There were 30,412 probe sets on the array chip. Left panel: The total number in each circle indicates the number of genes detected. Right panel: The number in each part indicates the number of genes detected except for overlapped part. (PNG) [file pone.0197165.s005.png]

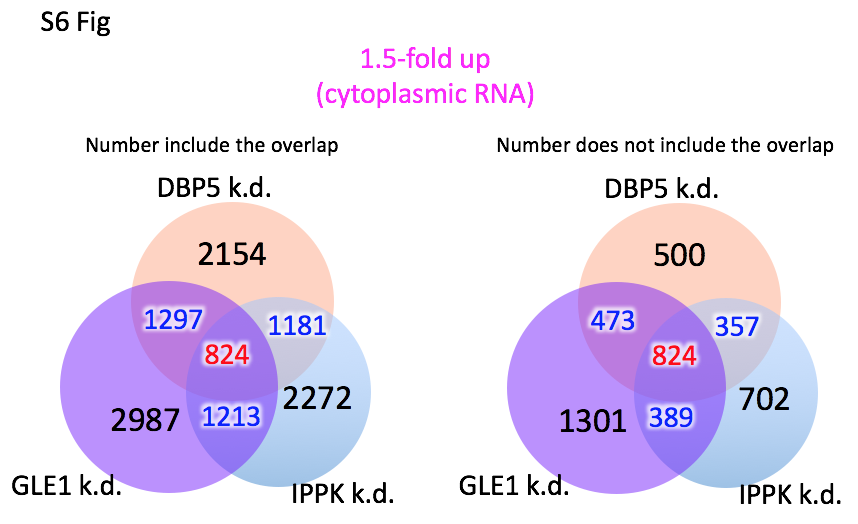

Supplement: S6 Fig — There were 30,412 probe sets on the array chip. Left panel: The total number in each circle indicates the number of genes detected. Right panel: The number in each part indicates the number of genes detected except for overlapped part. (PNG) [file pone.0197165.s006.png]

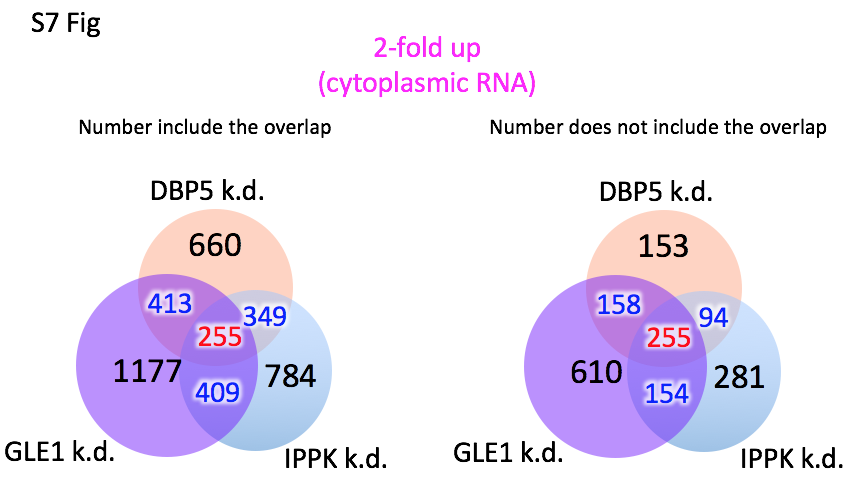

Supplement: S7 Fig — There were 30,412 probe sets on the array chip. Left panel: The total number in each circle indicates the number of genes detected. Right panel: The number in each part indicates the number of genes detected except for overlapped part. (PNG) [file pone.0197165.s007.png]

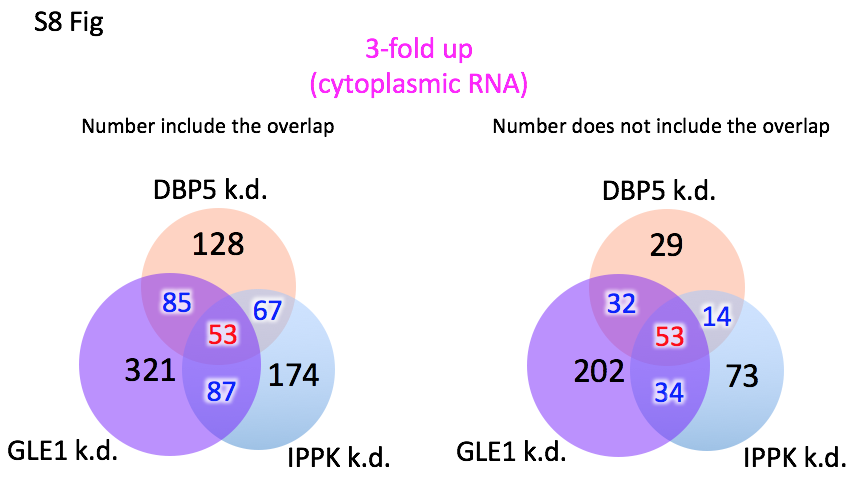

Supplement: S8 Fig — There were 30,412 probe sets on the array chip. Left panel: The total number in each circle indicates the number of genes detected. Right panel: The number in each part indicates the number of genes detected except for overlapped part. (PNG) [file pone.0197165.s008.png]

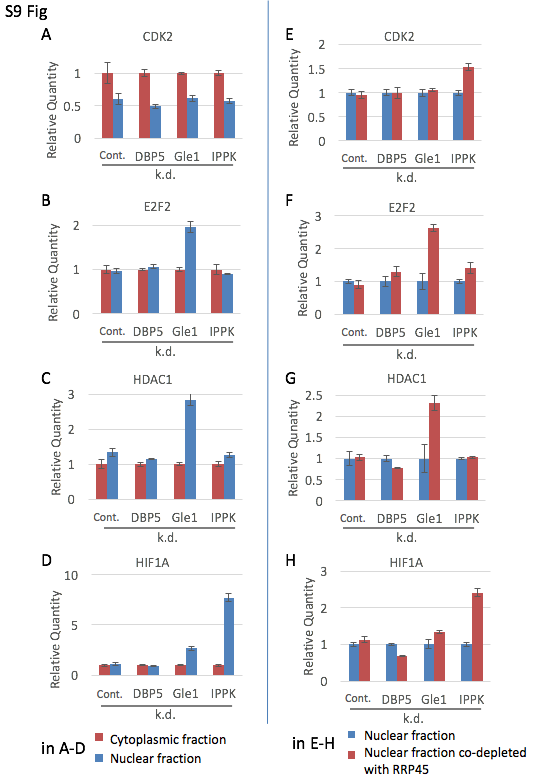

Supplement: S9 Fig — A-D, The cytoplasmic (red color) and the nuclear (blue color) mRNA expression level were measured and normalized with PGK1 by real-time PCR. A, CDK2, B, E2F2, C, HDAC1, D, HIF1A, The cytoplasmic mRNA expression level in each condition was set as 1. Each value is the mean with standard deviation of three independent experiments. Error bars represent standard deviations. E-H, The level of mRNA in DBP5, GLE1 or IPPK depleted condition in the nucleus (blue color) was compared with those of the condition co-depleted with RRP45 (red color). E, CDK2, F, E2F2, G, HDAC1, H, HIF1A, The mRNA expression level in the nucleus in each factor depleted condition was set as 1. Each value is the mean with standard deviation of three independent experiments. Error bars represent standard deviations. (PNG) [file pone.0197165.s009.png]

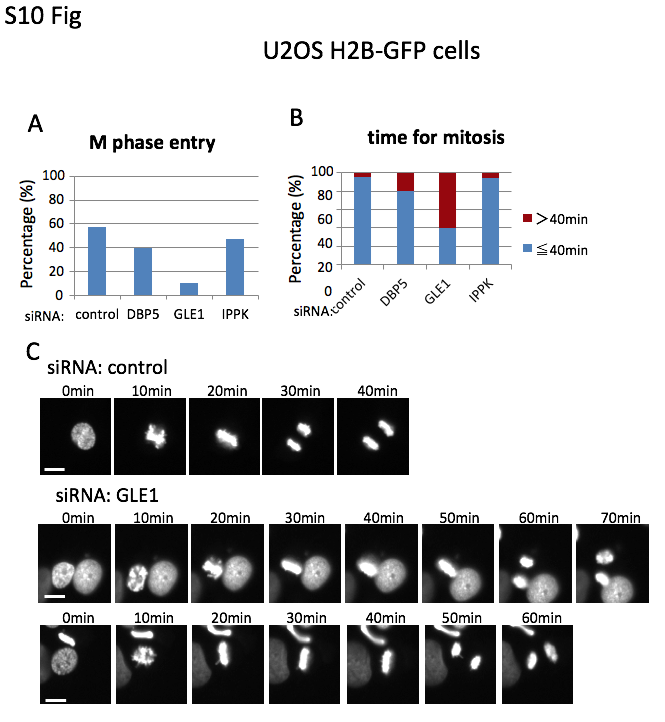

Supplement: S10 Fig — (A) The ratio of cells that could enter into the M phase. (B) The percentage of M-phase cells taking more or less than 80 min for mitosis. The numbers of cells counted were 126, 20, 10 and 33 in control, DBP5, GLE1 and IPPK siRNA-treated cells, respectively. (C) Representative successive live cell images for the indicated siRNA-transfected cells. Cells were observed 40–57 h after siRNA transfection, and the time was measured from M-phase progression by analyzing the recordings. Scale bar, 20 μm. (PNG) [file pone.0197165.s010.png]
